# Enhancement of YTHDF2 plays a protective role in acute IRI models through downregulation of TUG1 expression

**DOI:** 10.1371/journal.pone.0319605

**Published:** 2025-04-24

**Authors:** Hong-Fei He, Shuang Hou, Xiao-Ya Ma, Song-song Huang, Bo Yang, Jun-Kang Wang, Yuan Xu, Lei Tan, Hai-Yang Li

**Affiliations:** 1 Department of hepatobiliary surgery, Affiliated Hospital of Guizhou Medical University, Guiyang City, Guizhou Province, People’s Republic of China,; 2 Center for Energy Metabolism and Reproduction, Institute of Biomedicine and Biotechnology, Shenzhen Institute of Advanced Technology, Chinese Academy of Sciences, Shenzhen City, Guangdong Province, People’s Republic of China,; 3 School of clinical medicine, Guizhou Medical University, Guiyang City, Guizhou Province, People’s Republic of China,; 4 Department of Cardiology, Shenzhen Guangming District People’s Hospital, Shenzhen City, Guangdong Province, People’s Republic of China,; 5 Department of Pathology, Affiliated Hospital of Guizhou Medical University, Guiyang City, Guizhou Province, People’s Republic of China,; 6 Department of organ transplantation, Affiliated Hospital of Guizhou Medical University, Guiyang City, Guizhou Province, People’s Republic of China,; 7 Affiliated Hospital of Hangzhou Normal University, Hangzhou City, Zhejiang Province, People’s Republic of China,; 8 Innovation Center of Suzhou Nanjing Medical University, Suzhou City, Jiangsu Province, People’s Republic of China,; 9 State Key Laboratory of Reproductive Medicine and Offspring Health, Nanjing Medical University, Nanjing City, Jiangsu Province, People’s Republic of China,; 10 National Center of Technology Innovation for Biopharmaceuticals, Suzhou City, Jiangsu Province, People’s Republic of China; University of Pennsylvania, UNITED STATES OF AMERICA

## Abstract

As one of the major causes of acute kidney injury, renal ischemia-reperfusion is a common health problem in a series of clinical situations, including renal transplantation. Although the mechanisms of renal IRI have been widely investigated, effective strategies are still in lacking for its prevention and treatment. In previous study, we found that the down-regulation of taurine upregulated gene 1, a long non-coding RNA (lncRNA TUG1), markedly alleviated renal IRI through mitigating the cell inflammation and apoptosis. At meanwhile, YTHDF2, an RNA methylation reading protein, was identified as a vital player in IRI of distinct organs, however, not reported in kidney. We then conducted the current study on the function of YTHDF2 in renal IRI and its regulatory role to TUG1. Based on renal IRI models in vitro and in vivo, dramatical down-regulation of YTHDF2 was presented. Subsequently, exogenous perturbation of YTHDF2 was conducted and its protective effects on cell apoptosis were demonstrated in acute IRI exogenous. Furthermore, with the same model, it was indicated that YTHDF2 protein negative regulated TUG1 RNA via direction interaction. Since then, YTHDF2 was proved as a potential protector of renal IRI through restraining of TUG1. In further speculation, induction of YTHDF2 in IRI will possibly become a possible strategy to combat the pathological process post renal transplantation or other clinical conditions.

## Introduction

Kidney is highly sensitive to ischemia and reperfusion (IRI) [1], which occurred frequently after renal transplantation and has become one of the main causes of acute kidney injury (AKI) [2] and delayed graft function (DGF) [3]. These effects commonly result in acute or chronic immune rejection and ultimately elevated risk of failure in transplantation [3]. As a pathological state, re-supply of blood to tissues or organs after a period of ischemia will aggravate the long-term, even fatal dysfunction and structural damage in kidney [1,4–6]. According to International Registry in Organ Donation and Transplantation (IRODaT), increasing number of kidney transplantations were conducted in China and one million cases had been reached in 2022, which was 60 thousand more than in 2021. Unfortunately, the incidence and mortality of renal IRI kept increasing synchronously in past decades. In 2019, the death rate had reached about 20% among hospitalized patients [7,8].

Thus, great attention had been attracted in prevention and treatment of renal IRI to reduce the mortality in kidney transplantation. Various hypothetical mechanisms were then proposed to understand the pathology of renal IRI [9–11]. Basically, Inflammation and apoptosis were regarded as the key procedure in occurrence and development of injury in kidney [12]. As widely reported, induction of reactive oxygen species (ROS) level and risk of inflammation was triggered by a prolonged ischemia following with blood re-supply [1,13,14]. Consequently, cell apoptosis, autophagy, necrosis, and necroptosis were induced and led to further tissue damage [1,13,14]. In this context, panels of genes and pathways were suggested as functional players, such as mir-10a, AKT/mTOR signaling and TLR2&4 [9–12]. But unfortunately, clinical protection of kidney was still impeded due to the remained unclear and controversial mechanism of renal IRI. Since then, an urgent requirement was raised for further identification of the vital regulators in this program.

In our previous studies, a long non-coding RNA termed taurine-upregulated gene 1 (lncRNA TUG1) was indicated as a potential player in renal IRI. Downregulation of this molecule attenuated the inflammation and apoptosis in renal tubular epithelial cells induced with ischemia-reperfusion. In downstream, TUG1 RNA sponged miR-449b-5p, a microRNA targeting and repressing the translation of HMGB1 and MMP2 mRNAs [15]. Once TUG1 down-regulated, releasing of miR-449b-5p occurred and the repression on HMGB1 and MMP2 was then reinforced, from which the protection effects on renal cells was achieved [15]. However, the upstream regulator of TUG1 is still unknown in renal IRI circumstance, which is potentially applicable to prevent or rescue of kidney damage in clinics.

Interestingly, some reports on YTHDF2, a m^6^A methylated RNA reader molecule, has emerged in a wide panel of studies about IRI in distinct organs [16–21]. In most cases, this protein was indicated as the alleviator of IRI through mitigation of cell inflammation or apoptosis [19, 20]. In molecular level, its function was mediated through accelerating the degradation of target RNAs [16-18]. For instance, myocardial IRI was relieved by YTHDF2 through down-regulating BNIP3 via m^6^A modification [19]. Also, in ischemic stroke, hemorrhagic transformation post-reperfusion was relieved by matrilin-3 under the control of YTHDF2 [20]. Conversely, YTHDF2 was discovered as the driving force of ferroptosis in cardiac IRI via degradation of SLC7A11 [21]. Since then, the potential role of YTHDF2 playing in renal IRI becomes worth noting, while its interplay with TUG1 is also with great interests for validation.

Additionally, an in vitro model was frequently used in former studies of renal IRI. In this model, oxygen glucose deprivation/ re-oxygenation (OGD/R) was carried out on HK-2, a human renal tubular epithelial cell line. Similar strategies have also been widely employed in IRI studies of other organs, including cardiac, hepatic and cerebral [22–24]. With these OGD/R model, C1q/tumor necrosis factor-related protein 3 (CTRP3), CXC chemokine ligand 16 (CXCL16) and Drp1 molecules have been identified as the key regulators of IRI in heart, liver, or brain [22–24].

Therefrom, our current study was carried out to address the functional role of YTHDF2 in renal IRI based on the OGD/R model of HK-2. After visualized the dysregulation of YTHDF2 in renal IRI models, perturbation of this gene was conducted to clarify its function, especially to see the relationship with cell apoptosis. Subsequently, the regulation and interaction between YTHDF2 protein and TUG1 RNA was proved, while the effects on molecules downstream of TUG1 were also evaluated [25, 26]. With this work, new insights will be brought out to understand the regulation mechanisms of renal IRI, and to forecast the potential value of YTHDF2 in prevention and rescuing of kidney damages in clinics.

## Materials and methods

### Cell culture

Human renal tubular epithelial cell line HK-2 was purchased from Pricella Biotechnology Co., Ltd., Wuhan, China, and applied to establish the IRI model *in vitro*. This cell line was cultivated in Minimum Essential Medium (MEM) (Thermo Fisher Scientific, China) supplemented with 10% of fetal bovine serum (Pricella Biotechnology Co., Ltd, Wuhan, China) at 37°C with 5% of CO2. Cell split was performed at 1:2 ratio every two days.

### Establishment of the oxygen glucose deprivation/ re-oxygenation (OGD/R) model

HK-2 cells were seeded in six-well plates initially with density of 1 × 10^5^ cells/well. Once reached 80% confluence, serum free medium was applied to replace the FBS containing medium. 1.5ml/well liquid paraffin (Aladdin Biochemical Technology Co., Ltd., Shanghai, China) was added on the top of medium immediately and kept for 12 hours. After that, both medium and paraffin were removed. At meanwhile, cells were washed with PBS (Thermo Fisher Scientific, China) for 3 times and maintained for another 12 hours in FBS containing medium.

### Establishment of mice renal I/R injury model

All protocols of mouse modeling in present study were approved by the Ethics Committee on Animal Experiments of Affiliated Hospital of Guizhou Medical University.

SPF grade C57 Males were purchased from Beijing Charles River Laboratories (CRL) Experimental Animal Technology Co., Ltd. (Beijing, China) and manipulated for IRI modeling at 7-8 weeks age with body mass within 20-25g range. Before used, all mice were housed in the barrier environment for no less than 1 week before experiment. The following conditions were guaranteed for all mouse: room temperature in 20–25°C, 50–60% relative humidity, good ventilation, free access to food and water, and a 12-hour light/dark cycle.

In construction of in vivo model of IRI, 5 mice were employed for repeats. At beginning, anesthesia was performed via intraperitoneal injection of 30 mg/kg sodium pentobarbital. Midline incision was created on the abdomen, and then the right kidneys of each animal were resected immediately as control groups. On the other side, remained left kidneys were subjected to a 45 min occlusion using non-traumatic vascular clamps on renal pedicles. After clamps released, mice were maintained in warm and moist condition for a 24-hour reperfusion [15,27]. Occlusion and re-supply of blood were confirmed visually based on change of tissue color. 24 hours after surgery, anesthesia was carried out in same condition for the second round. The renal tissues and blood samples were then collected one-by-one for further assays.

### Gene expression perturbation of YTHDF2

To change the expression level of YTHDF2, plasmids and corresponding lentiviruses were prepared commercially by GeneChem, Shanghai, China, including one for overexpression (46928-X1), three for knockdown (#86717-1, 86718-1 and 86719-1 of LV-YTHDF2-RNAi) and two corresponding controls (CON335 for overexpression and CON313 for knockdown).

One night before lentiviral infection, HK-2 cells were seeded into 6-well culture dishes with density of 5 × 10^4^ cells/well. Viruses were applied into 2ml of FBS containing medium and reached the final titer of 2.5 × 10^5^ TU/well. 40μl/well HitransG (A & P) reagent was added to facilitate infection. Puromycin was subsequently used in 5μg/ml to select the cells 24 hours post-infection for 4 days. Remained cells were collected for expansion and further assays.

### Cell viability assay

Cells were inoculated in 96-well plates with density of 5000 cells/well. With OGD/R model established, cell counting kit-8 (CCK-8) (Bergolin Biotechnology Co., Dalian, China) solution (10μL per well) was added into the 96-well plates and incubated at 37°Cfor 2 hours. The absorbance was then measured at 425nm wavelength with Multiskan GO (Thermo Fisher Scientific, China). Cell viability was calculated as following:

Cell viability (%) = [A (test)-A (blank)]/ [A (control)-A (blank)] ×100%

A (test) － Absorbance of cells with adding CCK-8 and drug solutions.

A(blank) － Absorbance of media and CCK-8 solution, but no cells

A(control) － Absorbance of cells with adding CCK-8 solution but no drug solution

### Western blotting (WB)

The total protein samples were extracted from cells and tissue samples with RIPA lysis buffer (Solarbio Life Science, Beijing, China), and then kept in -80°C before use. Concentration of protein was measured with bicinchoninic acid (BCA) Protein Quantitation kit (Epizyme Biotech, Shanghai, China).

Western blot was performed routinely. The results were visualized with developer solution ECL Chemiluminescence Kit (Beijing 4A Biotech Co., Ltd, China) in chemiluminescent imaging system (Bio-rad, Hercules, CA, USA). Gray values of bands were quantified using Image-J software (National Institutes of Health, Bethesda, MA, USA). GAPDH was used as internal control for normalization.

The following primary antibodies were used: YTHDF2 (24744-1-AP, 1:4000), MMP2 (10373-2-AP,1:1000), Bcl2 (80313-1-RR,1:1000), Bax (50599-2-Ig,1:2000), and Caspase-3 (82202-1-RR,1:5000) and GAPDH (10494-1-AP, 1:5000). The secondary antibody used was HRP-Goat Anti-rabbit. All antibodies were provided by Proteintech, Wuhan, China.

### Quantitative reverse transcription-polymerase chain reaction(qRT-PCR)

RNA samples were extracted from cells or tissue samples with RNA-easy Isolation Reagent (Vazyme Biotech Co., Ltd, Nanjing, China). Reverse-transcription was conducted with PrimeScript™ RT reagent Kit plus gDNA Eraser (Perfect Real Time) (RR047A, Takara Biomedical Technology Co., Ltd., Beijing, China), or Hifair® miRNA 1st Strand cDNA Synthesis Kit (Yeasen Biotechnology (Shanghai) Co., Ltd., Shanghai, China) for microRNAs alternatively. TB Green® Premix Ex Taq™ II (Tli RNaseH Plus) (RR820A, Takara Biomedical Technology Co., Ltd., Beijing, China) was used in Quantitative real-time PCR. Three replicates were set for each reaction. Expression level was calculated using 2 − ΔΔCt method with GAPDH as internal control. Primers were synthesized by Sangon Biotech Co., Ltd. Shanghai, China and listed in Table 1.

**Table 1:** Primer sequences used in qRT-PCR analysis.

Name of primer	Sequences
YTHDF2-F	TCTGGAAAAGGCTAAGCAGG
YTHDF2-R	CTTTTATTTCCCACGACCTTGAC
TUG1-F	GTCCCCTTACCTAACAGCATC
TUG1-R	TCACTCAAAGGGCTTCATGG
GAPDH-F	ACATCGCTCAGACACCATG
GAPDH-R	TGTAGTTGAGGTCAATGAAGGG
miR-449b-5p-F universal miR-R	GCCGAGGCAGTGTATTGTTAGCTGGC provided in Hifair® miRNA 1st Strand cDNA Synthesis Kit
F forward, R reverse	

### Histology and microscopic observation of renal tissue post IRI modeling

The kidneys, either control and modeled ones, were fixed in 4% paraformaldehyde overnight at 4°C. Fixed tissue samples were then embedded in paraffin and subjected to tissue sections in thickness of 7 μm. H&E staining were performed based on routine protocol. PASM staining was conducted step by step as below. In brief, Fixation, Embedding and Sectioning. Photos of H&E or PASM Stained slides were taken under microscope (mix60-fl, Micro-shot Optical Technology Co., Ltd., Guangzhou, China) at 20 × or 40 × magnification. To defined the destruction of renal tubules, randomly captured images were proceeded via ImageJ software and the area occupation was then quantified with enlarged lumen in H&E staining or negative staining on PASM slides.

Immunostaining was also carried out on paraffin sections. YTHDF2 (24744-1-AP, 1:400, Proteintech, Wuhan, China) and Alexa Fluor^®^647 AffiniPure (711-605-152,1:1000, Jackson, China) were adopted as primary and secondary antibody in PBS dilution, respectively. After de-paraffin and rehydration, slides were subjected to heated sodium citrate buffer for 30 min of antigen retrieval inside a normal-pressure steamer. 1 hour of blocking was performed at room temperature in PBS plus 1% horse serum. Overnight staining of primary antibody was carried out at 4°C in PBS contained 0.5% Triton X-100 and 5% horse serum. 1.5 hours of secondary antibody staining was done at room temperature and avoid light. Images of YTHDF2 immunostaining slides were scanned and visualized in Olympus OlyVIA software. Intensity of YTHDF2 signal in random fields of view was quantified with Image J software, either.

### RNA immunoprecipitation (RIP)

The RIP assay was performed with IVDSHOW® RIP RNA-Binding Protein Immunoprecipitation kit (IVD Technology Corporation, Zhangjiakou, China). HK-2 cells grown in 10 cm dishes with number of 1 × 107 cells were harvested after rinsed twice with pre-ice-cold PBS. Cell pellets were resuspended and lysed on ice for 10min in 1mL Buffer A (1×) diluted with DEPC treated water plus 1% protein and RNase Inhibitor. Cell lysate was subjected to centrifugation at 14000g at 4°C for another 10min. 10% volume of supernatant was saved as input. 200μL protein A + G beads was pretreated for 4 rounds with Buffer A at 4°C and mixed with 5μg anti-rabbit-IgG (Proteintech, 30000-0-AP) or YTHDF2 rabbit polyclonal antibody (Proteintech, 24744-1-AP) as the IgG control and IP sample, respectively. The mixture of beads and antibody was incubated at 4°C for 2 hours and then washed with Buffer A and incubated with cell lysate overnight. Another washing with Buffer B was then conducted and beads were collected with magnetic block. Precipitated protein and RNA were extracted according to the user manual of the kit. Level of TUG1 RNA in samples was ultimately detected with qRT-PCR and normalized to the input.

### Prediction of mammalian N6-methyladenosine (m^6^A) sites

SRAMP, a public server accessible freely, was applied for prediction at http://www.cuilab.cn/sramp/. The RNA sequences of TUG1 (Gene ID: NR_152868.2) were retrieved from NCBI and uploaded in FASTA format to the server following the user instruction of the websites. Candidate sites were then exported with confidence level scored and graded.

### Statistical analysis

Data were presented as mean ±  SD or ±  SEM for qPCR, cell proliferation and Western blot (WB) strip gray statistics. Statistical significance was defined with student *t*-test and indicated with p-values. A value of *p* < 0.05 was considered statistically significant and marked with * , **, ***, **** for < 0.05, 0.01, 0.001 and 0.0001, respectively. All statistical analyses were performed with GraphPad Prism 9.

## Results

### Cell apoptosis was observed in OGR/D model of HK-2 cells *in vitro* and expression level of YTHDF2 was notably downregulated in OGD/R model

**Fig 1:**
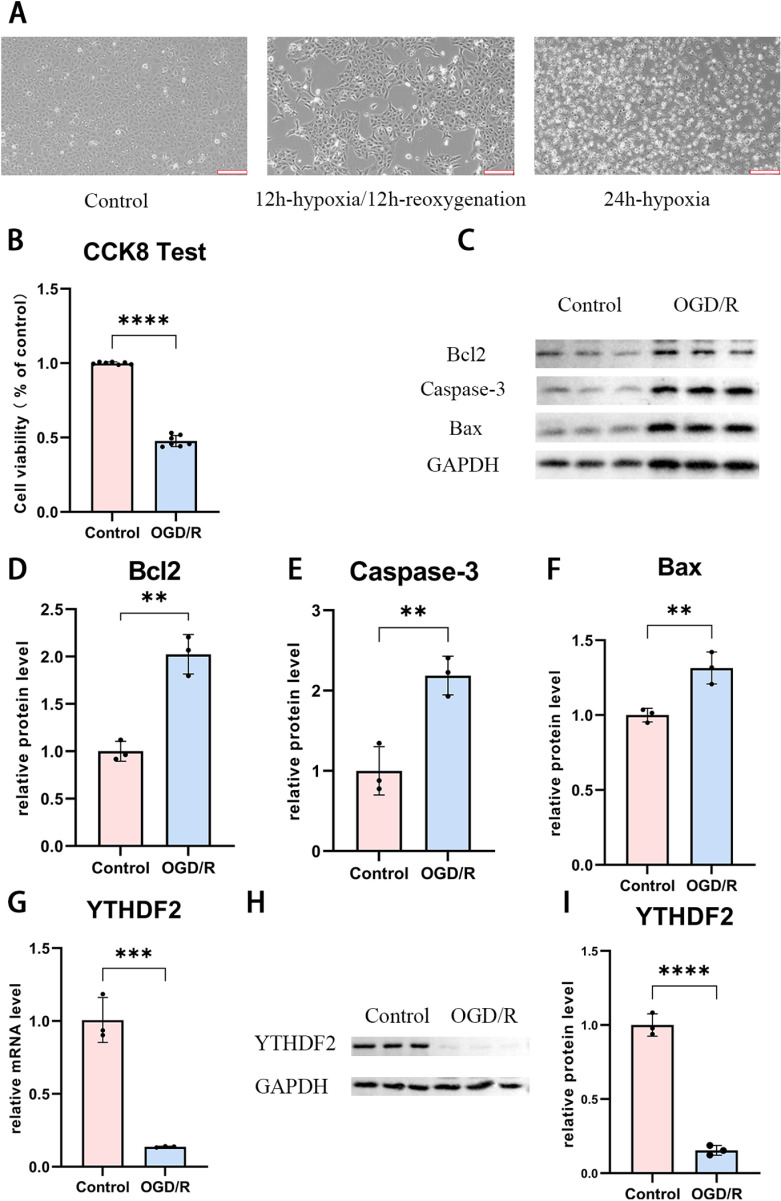
Construction and Validation of OGD/R model of HK-2 cells *in vitro* and a notable downregulation of YTHDF2 gene in OGD/R. (A) Microscopic images of HK-2 cells of OGD/R model. Left, control cells cultured in normoxia condition; middle, cells cultured in 12 hours of hypoxia and 12 hours reoxygenation; right, cells cultured in 24 hours of hypoxia but no reoxygenation. (scale bar =  200μm) (B) Viability of cells in (A) detected via CCK8. (C) Western Blot of apoptosis related proteins BCL2, Caspase-3 and BAX. (D-F) Quantification of the protein level in (C) by ImageJ software for (D) BCL2, (E) Caspase-3, and (F) BAX. (G) mRNA level of YTHDF2 in HK-2 cells of OGD/R detected by RT-PCR. (H, I) YTHDF2 protein detected in HK-2 cells of OGD/R by (H)Western Blot and (I) quantified base on the gray value of bands. Data was expressed as the mean ±  SD of three repeated experiments. **P <  0.01, ***P <  0.001 and ****P <  0.0001 vs. the control group. (n = 3 for cell experiments).

As the starting of current study, OGR/D model was constructed *in vitro* to simulate the injury of kidney cells under IRI. Effects were firstly monitored based on cell morphology under microscope. As the control, cells were cultured for 24 hours in normoxia and the culture surface was fully covered (Fig 1A, left). However, only 50% confluence of ells was reached after 12h-hypoxia/12h-reoxygenation (Fig 1A, middle). In the alternative control, viable cells were rarely remained post 24h-hypoxia (Fig 1A, right). More than 50% loss of HK-2 viability was also revealed using CCK-8 assay in the same condition of hypoxia/reoxygenation (Fig 1B). Significantly elevations were indicated for apoptosis related proteins, including BCL2, Caspase-3 and BAX, as visualized in WB (Figs 1C-F). Retardation of cell viability and activation of apoptosis suggested the success in model construction of renal IRI *in vitro*.

To find the clue of YTHDF2 functional in renal IRI, its expression was further monitored in established OGR/D model at both mRNA and protein level. ~ 87% decline of its mRNA was seen post hypoxia/reoxygenation (Figs 1G). Also, the protein level in modeled cells was measured as only 15% in control (Figs 1H and I). In one word, expression of YTHDF2 was significantly repressed in ODG/R model, which suggested its value for further investigation.

### Expression level of YTHDF2 was notably downregulated in IRI mouse model

**Fig 2:**
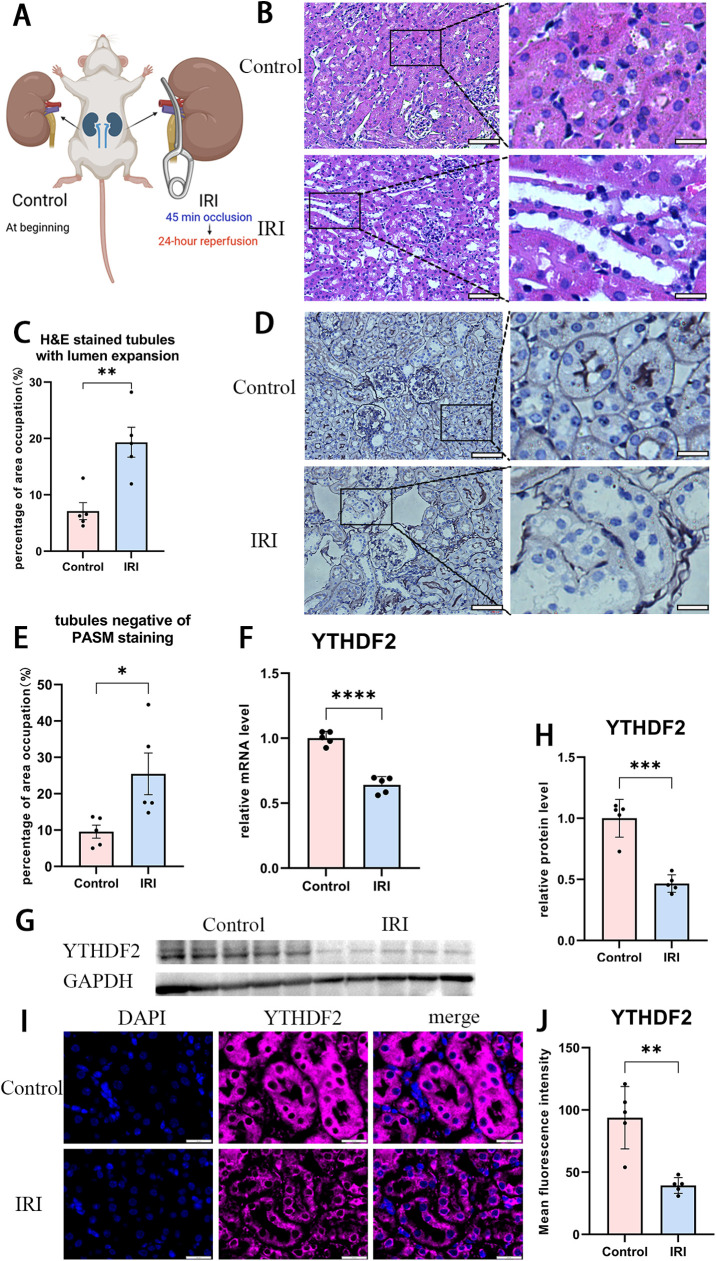
A notable downregulation of YTHDF2 gene in animal-based model of renal IRI. (A) Diagram showing the procedure of IRI model in mouse kidney. (B, C) Morphological changes were visualized in kidney with (B) H&E staining and then (C) quantified. (B) Representative images of control (upper left) and modeled tissues (lower left) were compared in 40 × magnification (Bar = 200μm). Renal tubules with regular morphology (upper right) or lumen expansion (lower right) were further magnified from left images (Bar = 50μm). (C) Frequency of enlarged lumens was counted within control and modeled renal cortex. For either group, 5 fields of vision were picked randomly from 5 kidneys. The result was presented as percentage of area occupied by tubules with lumen expansion in fields of view. (D, E) Microscopic imaging of basal membrane of renal tubules via (D) PASM staining and (E) quantified within cortex area. (D) Representative images of control (upper left) and modeled tissues (upper right) were compared in 40 × magnification (Bar = 200μm). Renal tubules with (lower left) or without (lower right) PASM staining were further magnified from upper images (Bar = 50μm). (E) Frequency of lumens absent of PASM was counted within renal cortex under 40 × magnification. For either group, 5 fields of vision were picked randomly from 5 kidneys. The result was presented as percentage of area occupied by tubules negative of PASM. (F) mRNA level of YTHDF2 in control and IRI kidneys by RT-PCR. (G, H) YTHDF2 protein level in control and IRI kidneys detected by (G) Western Blot and (H) quantified results based the gray value of bands. (I, J) Immunofluorescent imaging of YTHDF2 protein in (I) normal or IRI renal tissue under 60 × magnification (Bar = 20μm), while the staining intensity of was (J) quantified in 5 random fields of view for both groups. Data was expressed as the mean ±  SD of three repeated experiments. * P <  0.05, **P <  0.01, ***P <  0.001, and ****P <  0.0001. (n = 5 for mouse experiments).

To confirm the trend of YTHDF2 regulation in vivo, modeling of renal IRI was conducted in 7-8 weeks old C57 male mice (Fig 2A). When comparing with untreated control kidneys, enlarged lumens (Fig 2 B and C) and PASM negative renal tubules (Figs 2 D and E) were seen more frequently in modeled tissues. These signs indicated the destruction of renal tubules, and therefore, demonstrated the succeeded modeling of IRI in kidneys.

The expression change of YTHDF2 was then monitored in tissue samples with qRT-PCR and WB. Notably, IRI modeling led to a decline in mRNA around 40% (Fig 2F) and beyond 50% in protein (Figs 2G and H). As characterized with immunofluorescent staining, YTHDF2 protein was detectable in entire organ. In consistence with results of qRT-PCR and WB, intensity of YTHDF2 staining was significantly decreased in tubules of IRI, which were indicated with lumen expansion (Figs 2 I and J).

In one word, expression of YTHDF2 was significantly repressed upon IRI in vivo, which suggested its value of further investigation.

### Overexpression of YTHDF2 rescued the injury of HK-2 cells undergoing OGD/R

**Fig 3:**
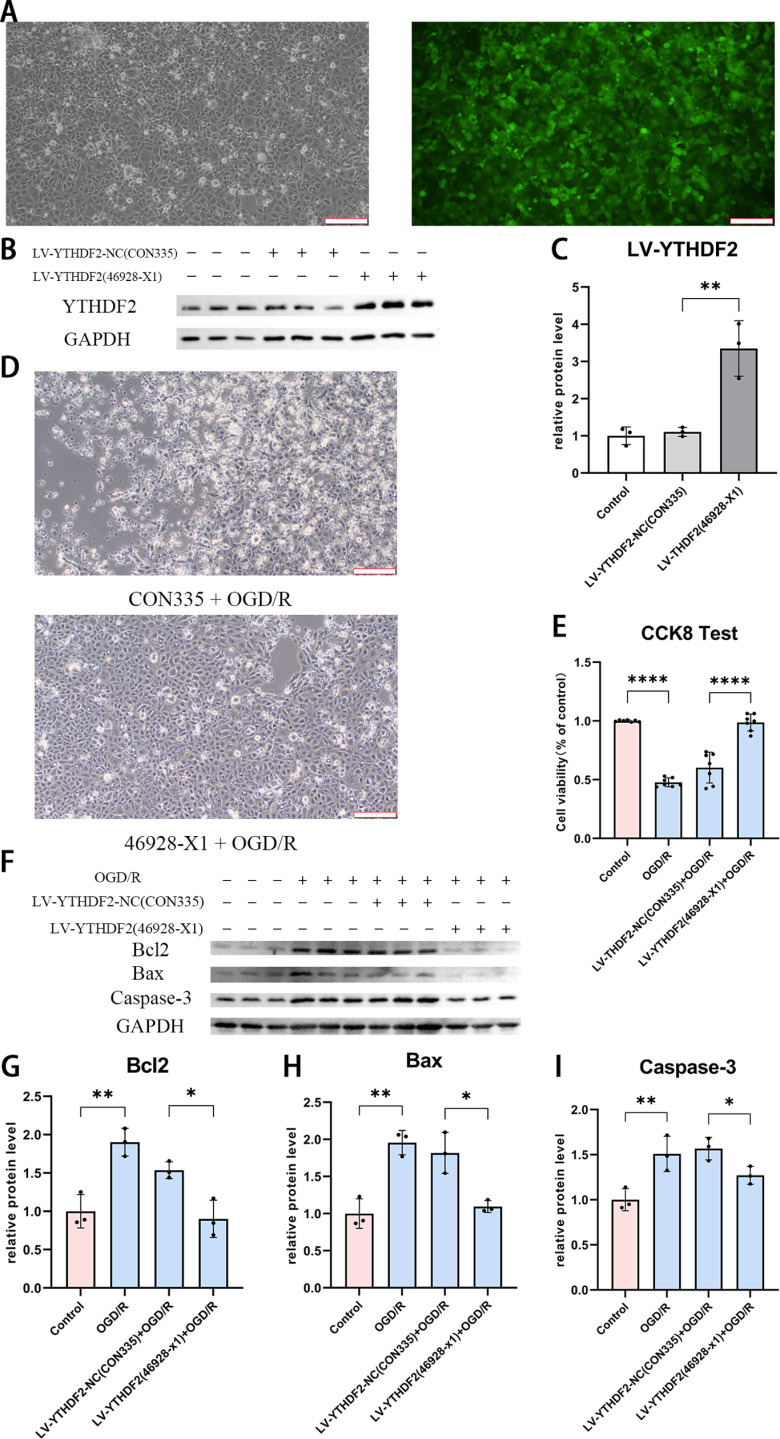
The effects of YTHDF2 overexpression in OGD/R model. (A) Microscopic images of HK-2 cells infected with lentivirus for the ectopic expression of YTHDF2 in bright field (left) and fluorescent field (right). The fluorescence signal was generated by the GFP protein in the lentiviral vector. (scale bar =  200μm) (B-C) Confirmation of enhanced YTHDF2 protein level with (B) Western Blot and (C) the quantified results. (D) Morphological characterization of YTHDF2 overexpressed and control cells in OGD/R model. (scale bar =  200μm) (E) Viability of cells with/without YTHDF2 overexpression in OGD/R model. (F-I) The trend of apoptosis related proteins indicted with (F) Western Blot upon YTHDF2 overexpression in OGD/R model and quantified respectively for (G) BCL2, (H) BAX and (I) Caspase-3. Data was expressed as the mean ±  SD of three repeated experiments. * P <  0.05, **P <  0.01 and ****P <  0.0001.

To further clarify the function of YTHDF2, overexpression of this gene was conducted with lentivirus. Success of infection was confirmed with GFP signal under microscope, which was incorporated in the lentiviral vector (Fig 3A). As quantified based on Western Blot, the elevation of YTHDF2 protein level reached about 3 folds in control cells upon normalization to GAPDH (Figs 3B and C).

After treatment of OGD/R, very little death and high confluence was seen in cells overexpressed YTHDF2 (Fig 3D, lower panel), while large portion of cell death emerged in cells with control infection (Fig 3D, upper panel). With CCK8 quantification, recovery of viability was also indicated quantitatively in YTHDF2 overexpressed cells underwent hypoxia/reoxygenation, rather than in uninfected cells or cells infected with control virus (Fig 3E).

At the potent courses of changed cell viability, expression of BCL2, Caspase-3 and BAX was then confirmed with western blot. Enhancement of expression was achieved in hypoxia/reoxygenation condition, however, was defeated when YTHDF2 overexpressed additionally (Fig 3F), in which the degree of down-regulation was calculated about 50% for BCL2, 50% for BAX and 20% for Caspase-3 (Figs 3G-I).

### YTHDF2 knockdown affected the viability of HK-2 cells in OGD/R model

**Fig 4:**
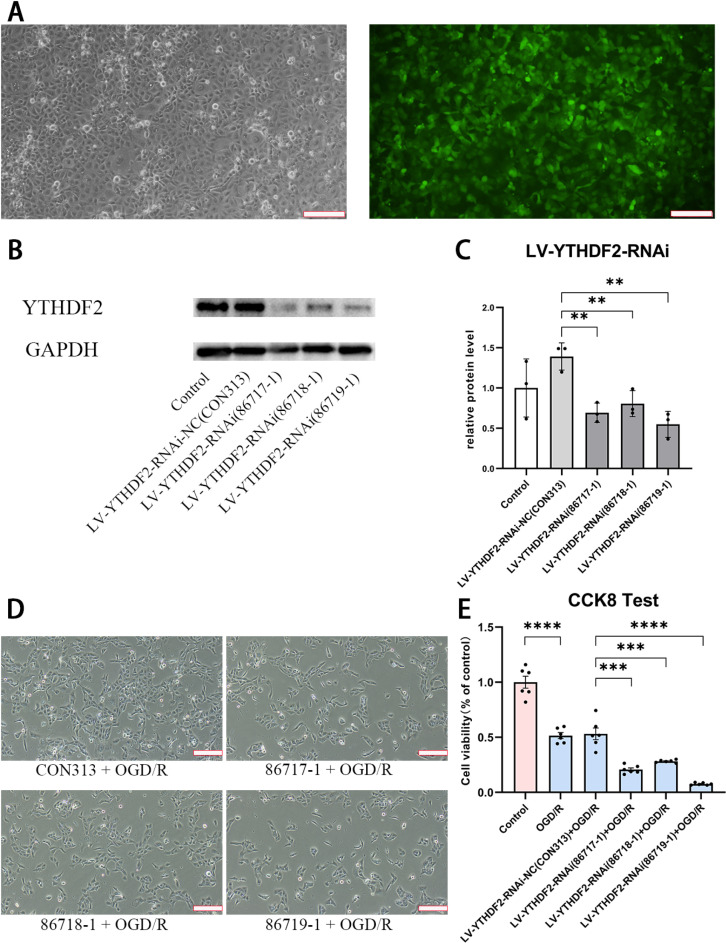
The effects of YTHDF2 knockdown in OGD/R model. (A) Microscopic images of HK-2 cells infected with lentivirus for the knockdown of YTHDF2 in bright field (left) and fluorescent field (right). The fluorescence signal was generated by the GFP protein in the lentiviral vector. (scale bar =  200μm) (B-C) Confirmation of impaired YTHDF2 protein level with (B) Western Blot and (C) the quantified results. (D) Morphological characterization of YTHDF2 knockdown and control cells in OGD/R model. (scale bar =  200μm) (E) Viability of cells with/without YTHDF2 knockdown in OGD/R model. Data was expressed as the mean ±  SD of three repeated experiments. **P <  0.01, ***P <  0.001, and ****P <  0.0001.

To confirm the outcome upon loss of YTHDF2 function, gene knockdown was performed additionally with 3 different shRNAs based on a GFP containing lentiviral vector (Fig 4A). The decrease of YTHDF2 protein level was confirmed with all shRNAs (Figs 4B and C). As visualized under microscope and quantified with CCK8, the impaired cell viability under hypoxia/reoxygenation was reinforced after administration of shRNAs to target YTHDF2 (Figs 4D and E).

### TUG1 RNA level was under the control of YTHDF2

Since YTHDF2 protein was reported as a m^6^A RNA binding protein, while lncRNA TUG1 were demonstrated as an important player in renal IRI by our earlier report, their potential interaction became worthy of evaluation.

**Fig 5:**
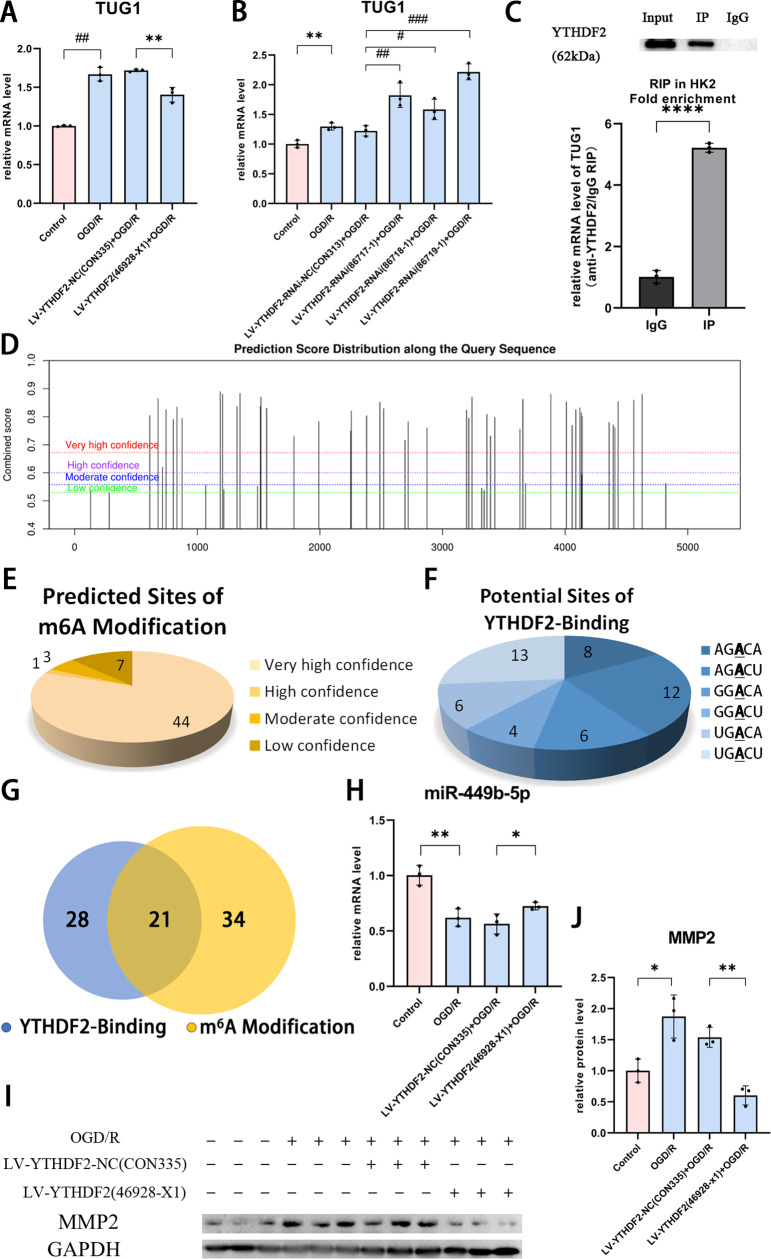
The regulatory role of YTHDF2 on TUG1 RNA in HK-2 cells under OGD/R condition. (A-B) The mRNA level of TUG1 detected by RT-PCR in HK-2 cells with (A) YTHDF2 overexpression and (B) YTHDF2 knockdown under hypoxia/reoxygenation condition. (C) RNA immunoprecipitation assay demonstrated YTHDF2 binding to TUG1 (****P <  0.0001). (D) The candidate m^6^A sites on TUG1 RNA predicted with SRAMP. (E) groups of m^6^A sites defined with their confidence level scored by SRAMP. (F) Potential binding sites of YTHDF2 recognized on TUG1 RNA chain based on the defined motif (A/G/U)GAC(A/U) (G) Number of candidate sites matching both criteria, potential of m^6^A modification and allowance for YTHDF2 binding. (H)The expression of miR-449b-5p in OGD/R-induced HK-2 cells with YTHDF2 overexpression were tested using qRT-PCR. (I-J) MMP2 proteins indicted with (I) Western Blot upon YTHDF2 overexpression in OGD/R model and (J) quantified respectively for MMP2. Data was expressed as the mean ±  SD of three repeated experiments. * P <  0.05, **P <  0.01, ***P <  0.001, and ****P <  0.0001.

To see the correlation of these two molecules, level of TUG1 RNA was measured under various conditions YTHDF2 perturbation plus OGD/R. Similar to our previous report [15], TUG1 RNA level was upregulated more than 50% in HK-2 cells under OGD/R and ~ 20% reduction was resulted once YTHDF2 overexpressed in addition (Fig 5A). At meanwhile, varying degree of up-regulation was achieved via YTHDF2 knockdown with distinct shRNA administrated in combination of OGD/R treatment (Fig 5B). In one word, abundance of TUG1 RNA was repressed by YTHDF2 protein.

Subsequently, to authenticate the potential interaction, RNA immunoprecipitation was then conducted with YTHDF2 antibody and measured with qRT-PCR. About 5 fold enrichment of TUG1 RNA was indicated in sample proceeded with YTHDF2 antibody when comparing with treatment of control IgG (Fig 5C), which confirmed the direct binding of these two molecules. The potential sites of interaction were then predicted in-silico. On one side, m^6^A modification is necessary for the binding, so SRAMP online platform was employed to highlight the modified sites. 55 candidates were then highlighted in total. These sites were distributed along the entire RNA chain of TUG1 (Fig 5D) and scored to 4 levels of confidence [26], including 44 sites as “very high”, 1 site as “high”, 3 as “moderate” and 7 as “low” (Figure 5E). On the other side, motif of (A/G/U)GAC(A/U) had been defined as necessity on target RNAs to form the complex with YTHDF2 protein [28]. 49 sites fitted this motif was marked on TUG1 sequence (Fig 5F). Overlapping of either list above, 21 sites was manifested to meet both criteria, potent in m^6^A modification and allowed for YTHDF2 binding (Fig 5G and Table 2), which will prompt depiction of interplay between YTHDF2 and TUG1.

**Table 2:** 21 potential binding sites of YTHDF2 protein on TUG1 RNA.

Position	Sequence context	Confidence
130	AUAGCAGACUCCUUG	Low
1187	UAAGGGGACUUCCUU	Very high
1515	UACACUGACAGACUC	Very high
1519	CUGACAGACUCCCUU	Very high
1566	GUACAUGACUUUGAA	Very high
2381	UACACUGACUCAGAG	Very high
2520	CAAGGAGACUUUGAU	Very high
2720	GCUGUAGACUGCUCG	Very high
2874	GAAAAAGACAUUGAU	Very high
3196	AACUUUGACAAAUCU	Very high
3241	AGCAUAGACUCCUAA	Very high
3340	GUACCUGACUGGCUC	Low
3655	CACUAGGACUCAAUG	Very high
4010	CAGCAGGACAGUUGG	Very high
4087	CUAUUGGACAUGAUU	Very high
4120	AUUACAGACUUCUUG	Very high
4131	CUUGAGGACAGGACA	Very high
4136	GGACAGGACAAACUU	Moderate
4359	GGUUUGGACAGUCUG	Very high
4560	UGGAGAGACUUGAAU	Very high
4628	CCCCUGGACUUUUCA	Very high

To see the downstream effects of YTHDF2, two molecules under the regulation of TUG1, termed miR-449-5p and MMP2, were monitored in HK-2 cells of OGD/R. When YTHDF2 expression reinforced, reduction of miR-449b-5p level was partially compensated (Fig 5H), and boost of MMP2 protein was restrained (Figs 5I and J). Considering the repression role of YTHDF2 on TUG1, these results were consisted with the regulation between TUG1 and its downstream molecules in renal IRI program.

## Discussion

Various investigations have been conducted on renal IRI to provide new insights of the pathological mechanisms [29–32]. Disease modeling of IRI is fundamental to identify new regulators and validate new programs. Although a series of renal IRI models had been constructed *in vivo* and *in vitro*, the cost and complexity are still the major concerns [33–36]. A highly accessible and applicable OGD/R in vitro model simply based on HK-2 cells was employed in former IRI studies and also our current one [22–24]. Apoptosis was commonly induced and addressed to the loss of specific AMPK in renal tubular epithelial cells [37]. Generally, Bcl2, Bax and Caspase family members, especially -3 and -9, were employed as the indicators of apoptosis in IRI models, which was also hired in this study [37–40]. In technics, hypoxia condition had been optimized as 12-hour application of liquid paraffine. Based on such condition, retardation of cell viability and induction of apoptosis were clearly exhibited at the initiation of current research. This is well agreed with previous studies applying similar models [34,35].

For the first time, functional role and distribution pattern of YTHDF2 protein were demonstrated in kidney of IRI based on this work. Before our report, importance of this gene had emerged in heart and brain IRI already [19–21,41,42]. Generally, this protein represses apoptosis and alleviate the tissue damage [19,20]. In current study, a significant reduction of YTHDF2 expression was seen post hypoxia/re-oxygenation both in vitro and in vivo. Also, this protein showed dominant abundance in cortex rather than medulla. Via further overexpression and knockdown, protective role of YTHDF2 was demonstrated. Activation of apoptotic genes in OGD/R cells was defeated with enhanced YTHDF2. These Phenomenon was well consistent with majority of earlier reports on IRI of organs. New insights provided in current work will show clues for the prevention and treatment of kidney injury in transplantation.

Discovering downstream effectors of YTHDF2 protein is necessary for either understanding of regulatory mechanisms or facilitating its clinical application in the future. However, for this new regulator declared in renal IRI, no target molecule has been identified under its control. In earlier reports, YTHDF2 was described as a m^6^A methylated RNA reader and usually plays its function via direct binding and accelerating the targeted RNA degradation [16–18]. Coincidentally, lncRNA TUG1 was indicated to promote renal IRI in our previous study, which is opposite to the role of YTHDF2 in renal IRI. Opposite trends were also proved in abundance of these two molecules in OGD/R circumstance. Therefore, the potential interaction between YTHDF2 and TUG1 was taken into our consideration.

Following the exogenous perturbation of YTHDF2 in renal cells, mRNA level of TUG1 was changed conversely, which suggested YTHDF2 as a suppressor of TUG1. This perspective was reinforced with the RIP data, which is strongly supportive to the direct binding between these two molecules. Furthermore, a list of 21 potential sites for YTHDF2 binding was predicted in TUG1 mRNA via in-silico pathways. On these sites, great consistency was exhibited between the potential of m^6^A modification and the defined binding motif of YTHDF2 on target RNA. Mapping of exact binding sites between YTHDF2 and TUG1 will possibly be benefit from this prediction. Additionally, the regulation of YTHDF2 on two downstream effectors of TUG1, miR-449b-5p and MMP2, showed that YTHDF2 can be integrated into the network centering of TUG1 as an upstream controller, rather than interplays with TUG1 solitarily. These findings presented TUG1 as a new target of YTHDF2 and gave clues for further discovery in related programs of renal IRI.

In conclusion, for the first time, our present study demonstrated that YTHDF2 is a protector in renal IRI and lncRNA TUG1 as its target molecule. With binding of YTHDF2 on some certain m^6^A sites of this target RNA, repression was imposed not only on TUG1 itself, but also to the correlated network. This study provided new insights into the molecular mechanism of renal IRI, and suggested the YTHDF2/TUG1 axis to be a potentiated target for future therapies.

## Supporting information

S1 File Supplementary document. (ZIP)

S2 File Tissue picture. (ZIP)

S3 File WB original blots. (ZIP)
