# Supplementary material for: Enhancement of YTHDF2 plays a protective role in acute IRI models through downregulation of TUG1 expression
Source: PLoS One. 2025 Apr 24;20(4):e0319605. doi: 10.1371/journal.pone.0319605 (PMC12021219; doi:10.1371/journal.pone.0319605)
Supplement: S1 File — (ZIP) [file pone.0319605.s001.zip › Supplementary Document 1.docx]

**Supplementary Document 1**

>NR_152868.2 Homo sapiens taurine up-regulated 1 (TUG1), transcript variant 5, non-coding RNA

GAGCGACGCA GCCGGGACGG UAGCUGCGGU GCGGACCGGA GGAGCCAUCU

UGUCUCGUCG CCGGGGAGUC AGGCCCCUAA AUCGAAGAAG CCCUGGCGCG

CCCUCCCCCC CUCCCGGGUC UGAUAGC**Aga cU**CCUUGAAA GCAGGGUCCU

UGUUUAGUGC AUCUUUGCCC ACAUACACCA CAACAUAUCA AGAUGCAUUU

AUUAGGAAGG AGGAGUUUAG AGAGCAGGCU AUCAGAAUAA CCACUCAUCC

UGUGCCUCCU GAUUGCUGAG UGUUCACCUG GACCUUC**Uga cU**ACCUUCCC

UGUGCUAUUC CAUCAGCCUA CAGACCUGGU ACCUGGAUUU UUGCCCGAGA

UGAUUCCUAC CACCUUACUA CUGACGA**Aga cA**CCCAUUCC AGUGGACCAC

UGUGACCCAG GAGGCAUUCA GCCAUCAUGA UGUGGCCUUU ACCUCCACUC

CUGUCUUGUU CUACCCAGAU UCAGCACAGC CCUUUAUAGU GAAGUCAGAG

UCCUCAAGCC AAAUAGCUAA AGCUGUUUUA UCACAACAAA GGCCUAGUUU

GUUCCAUGAG UGUGCAUUUC AUUUCUUCAG UUAAAGCCUU CAG**AgacA**CA

CAAUAAAUUU GGACCAGGGG AUUUUUUAGU UAUUAAUGCU CUCUGAAGAA

AGGCAACAUC UUUUUGAGAG CAGCAUUGGA CCACACCCCA CAAUCUCAAA

UGAUUGAAAU UCAUGAACAU CUAGGAUCCC GUGAAGGUCA CUGGACCCUG

UUUUUUCUAC UUCAAAUCCU GUAGUAGCCU ACUGAAUGAG AAAACAUAUU

CUGACCCAUU GGGAUCAAAU CAAAGGCACA GUGAACUCCU CAUAGCAUCU

UCUUUGGAAU UACUCAGGAA CCAGAACUUU UUACACAAAU GUAAGAAAUU

CUACCAAGGA GUCCCCUUAC CUAACAGCAU CUCACAAGGC UGCACCAGAU

UCCAGAAAAG GCUUCUCUUG AUACAUCAAG GUAGAACCUC UAUGCAUUUU

GUGACCGACU UAUUCUUAGA UCAUUGGUUU UCCAAAGGCU UUGUGGCCAU

GAAGCCCUUU GAGUGAAAAC UGUGCAGAAG CCCAGAGUAA AAGUGAAGCU

GCUCUGGAUG AAGUAGUGAA GCAAGAGUAG GGGCCUGAAU CCUGCUACAA

CUAUCUUCCU UUACCACCGU GG**UgacA**CCU AAGG**GgacU**U CCUUACAACA

CCUUGAACUC UUCCGAACAC AGUUUGAAAA CCACUGCCCC **AgacA**GCAAU

AUGUUUGACC UGAAUGGCAU UCCAAUCUUU UCUGUACCUC CACUCAGCAC

AGUUCAUGUU CAGUAGAUGC UGAACAUUCU UAGAAAUACU GUGUGUGAAC

UUAGAAAAGU GCAAGA**Agac A**GGCAUGUCU UUGACCCCAG GAAUGAUCAU

UUGCUGAAGA UGGUGUCAAG UGAACCUAGA UUAACAGCCC UCCACUCCAG

AUGGAUAUCC AGUGAUUCCU AGAAUGGGAU AUAGCCAGAG AACAAUUCUA

UGCACCCUAC AC**UgacAgac U**CCCUUAAGC AACACCAGAU GCUCUACUGG

UACUUGAAGU ACA**UgacU**UU GAAGUCUUGA CCCUCCAUGA AUACCUGAAU

UAUCAGCAAG CGGGUUUUGA AGCUGGUGCC UCAUUGAGGC CAUAUUAGAG

CAACUUGUAC AUUUGACCUC UUGUUAUCAG CCAUGGUACU CUACUUCGUG

UGCAAGAGAU AACUAUGAAA GCCAAAUUCA AAUACUGGCA ACAUUUCCUA

AAGGGGCUCA AUAUCUAUCA UUCGUCUUCU UUUCCAAACU ACACAUCACU

GUA**UgacU**CA ACCAGUAGCA GUUAUAUUGC CCCUUGGUUU UUAUUCAGUU

UAACUACUGU UUCCAAGAUA AAUGAGCUAA UAAGCUUUAA AAAAAAAAAA

AAAAAAGGCU GAAUUCUUUU UUCUUCAUCA CUGGCAUAUC UGCCUAUUCU

CCAGAAUUAU UA**UgacU**AUU CAGCUCACUU UAACAGUUGA ACUUCAAGCG

ACAAUCUUUG AACACCCCUU CUCAUGUGAU UUAAAAUGAA ACCAUUUGGA

AAAGUUUCUU CUAGCCAGUA AUAGAUUUUU UUUUUAAUUG CUCUGCCUUG

UGCCGAGAGA UGUUCUUUUA AGAUGAAUCU UUUGAUGUCU GAUACCACCA

AAUAUAGGUG GUAGGGAGAG UUGGAGGCUG GCCCUUUGAG CAGGCCAUUA

GCUUACUUGC UGGGCAUUUC CGAUAGCUUA UUGCCUACCU UUUUGCUGGA

AACAAACUGA UUUGAAAAAC AAAAUCUAUG A**AgacU**GCAG CUAAGGAUUU

UAUCGGU**Aga cU**UAAGAGCU UUUGUCCUUG UGGAUAUUUU AGUGGAACCA

CAUCAGUCUC AAUACUGUCA UUUUACAC**Ug acU**CAGAGCA GC**UgacU**UCA

UUCCUUGCCA UGAUAUAUAU UUAAGGCAGG CAUUGUAAC**A gacA**UAA**Aga**

**cA**ACUUAUCU GUUUCAGCAG GAAGGAUUCA GUUUAUGAAC UCUCAGACCA

GAUCAUGUUG AACAAGG**Aga cU**UUGAUGUG UGUCAUGAGA AAACUCAUUC

UUUACUUCCC AGUCAAUUUA AAGGCCAGCU AUCCUGAGCU ACUCGAAUGA

AUGCACUGGU UAAACAUUGG AAAUAGUUUG UUUAUAUCCU UGUCUCUCUC

UAGGCCAAUU GUGAUUACA**U gacU**CGACUC UACAUCUCGU CAAACAAGGC

CUAGGUCUGG UUGCUGU**Aga cU**GCUCGCCC UCAACAAAUA AAAUCUGGU**U**

**gacU**AGCCUC CUUGUAUAUA CAACUAUUAU UUGUUAAGAA GAAAUUAUCG

UCAAUUUUCU ACUACCUUCC AAUUGUCAGC UCUUUUUUUC CUCUCUGGUU

UUUCCUAUAC UUUACAGAAA A**AgacA**UUGA UCUAUACUGC CAUUCCCUCU

AAUCCUGCCA UACUCAGUCA AAAGGAA**Uga cU**UAAGAUGA AGAUGAUCAU

CUGCUCGAGU CUAAAAUAUA CAUUGUAUAU AAGAAUUGGU GAUUAGAAAA

GCAAAAAACC UAAAACUUAA AUCUAGGAGU CUGUAUACUG UCUCCAUGUC

UCCAUGCCUC AGAUCUCAUC UAAAUCUUUG AACAGCACCA UUCAACCAAU

CUGAGGCCU**U gacU**UGCUUG UAAGAUGAUU CUCAGAGAUC GGCUGAGUUA

AAAAAGAUGA CGACUUGAUU ACCAAAGAAA GUAGGGCCAA CUU**UgacA**AA

UCUGGCUCUG CUGACCCUGU CACUCCCAGA UGUAGCAU**Ag acU**CCUAAAC

AGAACCUCAA GUCUGAUUGA GGAUAAGGCC UUCUCCUGAG CUGAAAGUUC

UUUGGCAGAU GAGCAAGAAA CUGAAAGCUG AUGUACC**Uga cU**GGCUCUGU

AAGAUCAGAA AACUGUAUCC AGAAUAAGCC CUAUGGAUUA ACCCCUGAGU

ACCCAGAGUA AAAACUAAUU UACAGAACUU CCUUAUUGAU CUGCUGGUUC

UUCCAGAUCA UAUUCUGGCU AUUGGUAUGG CUGGCCUUUC UGAAGGUACC

CUGCUUGUCU AUUUUCC**Uga cU**CAGCUCUU GCCUGCCUUU UUCACAUGUU

GCUGCAAUU**A gacU**CACCGU GA**GgacU**ACA GUCAAUUUCA GUCUAUCUUG

UGCCCAAUAC AACAAGGAUU UUUAAUAGUA ACAACCCACA CCUCACCCAC

UA**GgacU**CAA UGUUCACAAC AGGAAGGACC AUUGCUGCAU ACUCCUUGAC

CAGCAACUUU UUUGAAGAUA UUUUUAAGUG CAGAGUAGGC CUCUAUUCCU

GUAUGUAAUU GUUCAUUUUC AGCACCUGGA ACCUCAUCUA UCGGGUCUGG

AAGGAAUACA GCAGUUCGAA AGCCGCGUCC AUUUCUCUCC UUCAGUAGUG

CAGAAAUGAG UCCGAUUCAC CAGUACACAC AGAACUGUAC CAGUUCAACC

UAGCAAAAGA AGAAAAGUUU CCACUGUACU UAAAAUUUAC AGC**UgacU**CA

AAUUGCCUCA CAGAAUUAUU UGAUGUAGAA GGCUAGUUGU CUUACUUCAG

AUCAGCA**Gga cA**GUUGGGCU CUC**AgacU**CA UGACCACUGA GUUUGCUUGU

GUUGAAACUG UGGUUUCAUC CAACAUAUGC UAUU**GgacA**U GAUUAUUAUU

CCAUUCAAAU GGAUUAC**Aga cU**UCUUGA**Gg acAGgacA**AA CUUAUCUCUC

AUGGUGUUUU UUUAGAAUAC UUUUAUAACC AAGGAAGAAA CCAUGCCAGC

UGUUACCAUU CAACUUCUUA AGCAGAGAUU AAGCUUUUUC AUAUCUGUUC

UUAUCCU**Gga cA**UCAGUAGU UUUUAAUUGC CCAGCAUCCG UUCCAUCUUG

UAACAACUCC CUGAUGUUUC UUAAAACCAC CUCUUCCUAU UUUCAGUCUG

UGGUUU**Ggac A**GUCUGACCC AACCUUGAGC UUUGUGGGUG AACAUGUAAU

UCAGACCUCA UCAAUCAGCA AAUCCAUCUG AACUGUGGAG GAGAAGCUCU

CUUUACUGAG GGUGCUUUAG CUUUGUAGGA UGAAAACCUC AAACUAACAG

GGCCUACCAU GUAGAGAAUG AAGCCAGUGC AGGGGAAAGC AGAGCCAAAA

UAUGGAG**Aga cU**UGAAUCCU GA**UgacA**GCG UUUGUGCCCC UGGAUCCAAC

CGUGCCUGAA GCUAGAAUAU CCCCU**GgacU** UUUCAGUUAU GUGAACCAAU

AAAUACCCUU UUUUGCUUAA GUUACUUUGA GUUGGGUUUC UGUUACUUGA

AAUUGAAUCC ACACUAAUAU AUCUACCAAC AUUG**AgacUU gacA**GAUCCA

AGUAUUUAUU AAGCUAGAGG UCAUGGUCAC UGAAAUUACU UUCCAAAGUG

GA**AgacA**AAA UGAAACAGGA ACUGAGGGAA UAUUUAAGAU CCCACAGAAG

CGUAAAAA**Ug acA**UGGUAGA AAGUAAUAGA AAACCUAAAU GUCUGUCAUU

AAAGGAUAGG UUAAGGUGUG GUUCAGCCAU AUAGGAAUAU CUCGUAUCUG

UUAAAAUGAA UAAAGUACAU UCAUUGUGUA UGGAAAAAUG GCCAUGAUAC

AUUAGGUGAA ACAAGUUAUU AAUAGAAAAG UGUACAGUGU GAACUCAUUU

UAAAAUGUGU GUGCUUAUGU UUAUAAAUGC AUAGAAAGGU CUAUUCACAG

CUUUCUUUGA ACAGUGUAGA UCACAUGAAA CUUUCAACUU UAUACAUUUC

UGUAUUAAUA UUUUACACUA CCCACAUUAU UUUUAAACUU UAUUUUAAAU

AAAGAAUUUU UAAAAUUAAA
