# Supplementary figures and images for: Enhancement of YTHDF2 plays a protective role in acute IRI models through downregulation of TUG1 expression
Source: PLoS One. 2025 Apr 24;20(4):e0319605. doi: 10.1371/journal.pone.0319605 (PMC12021219; doi:10.1371/journal.pone.0319605)

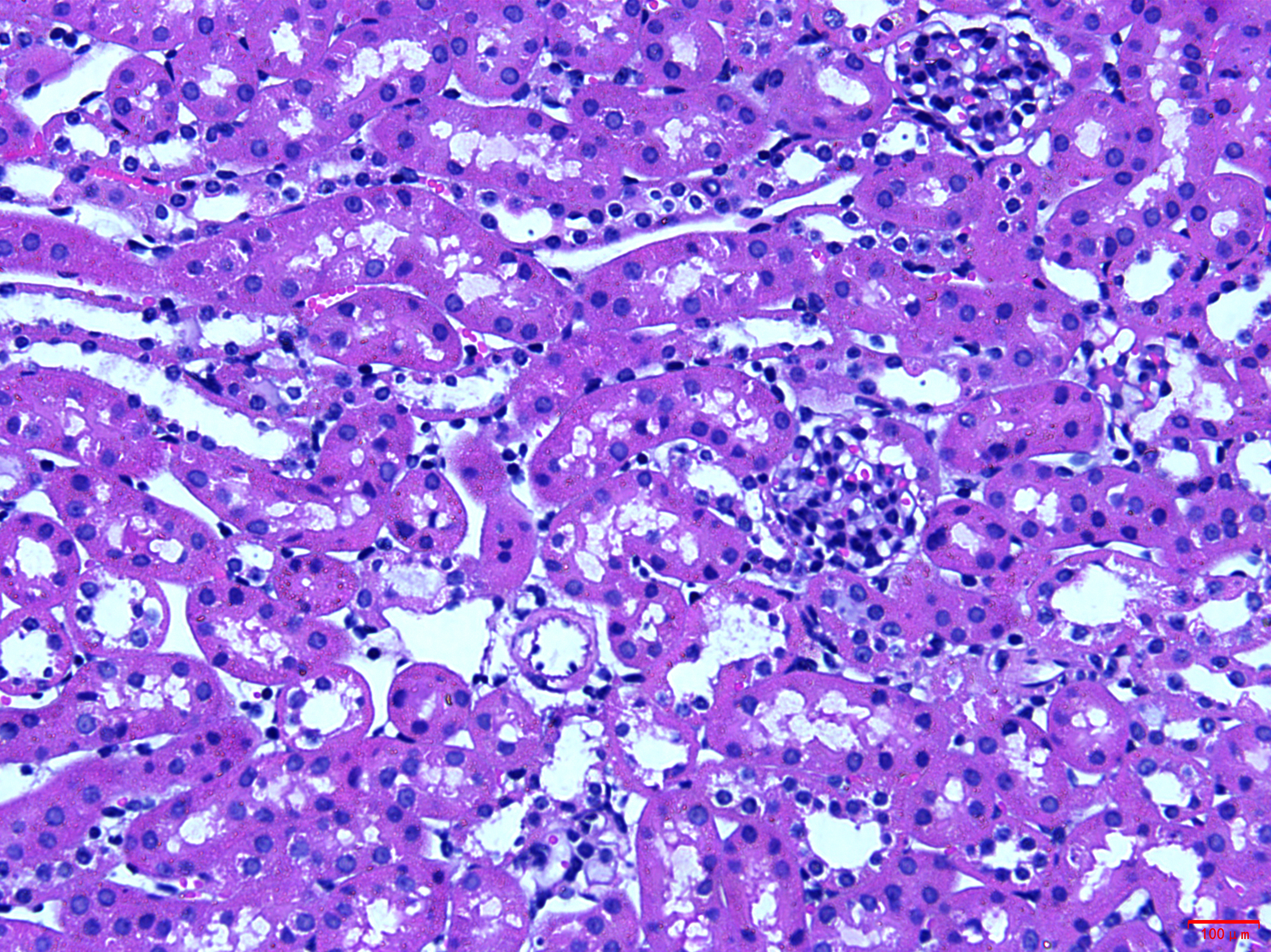

Supplement: S2 File — (ZIP) [file pone.0319605.s002.zip › Fig 2B low.jpg]

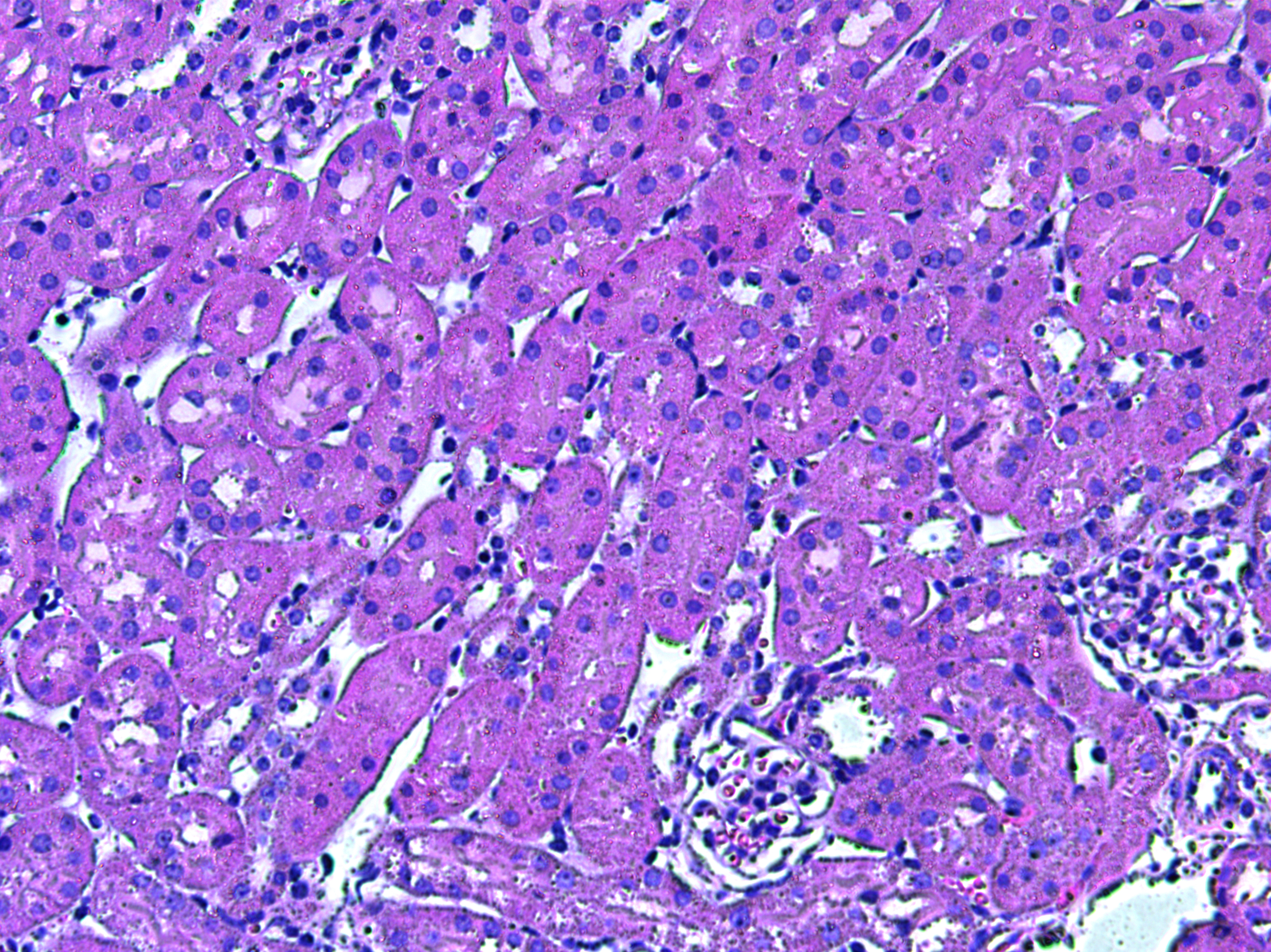

Supplement: S2 File — (ZIP) [file pone.0319605.s002.zip › Fig 2B up.jpg]

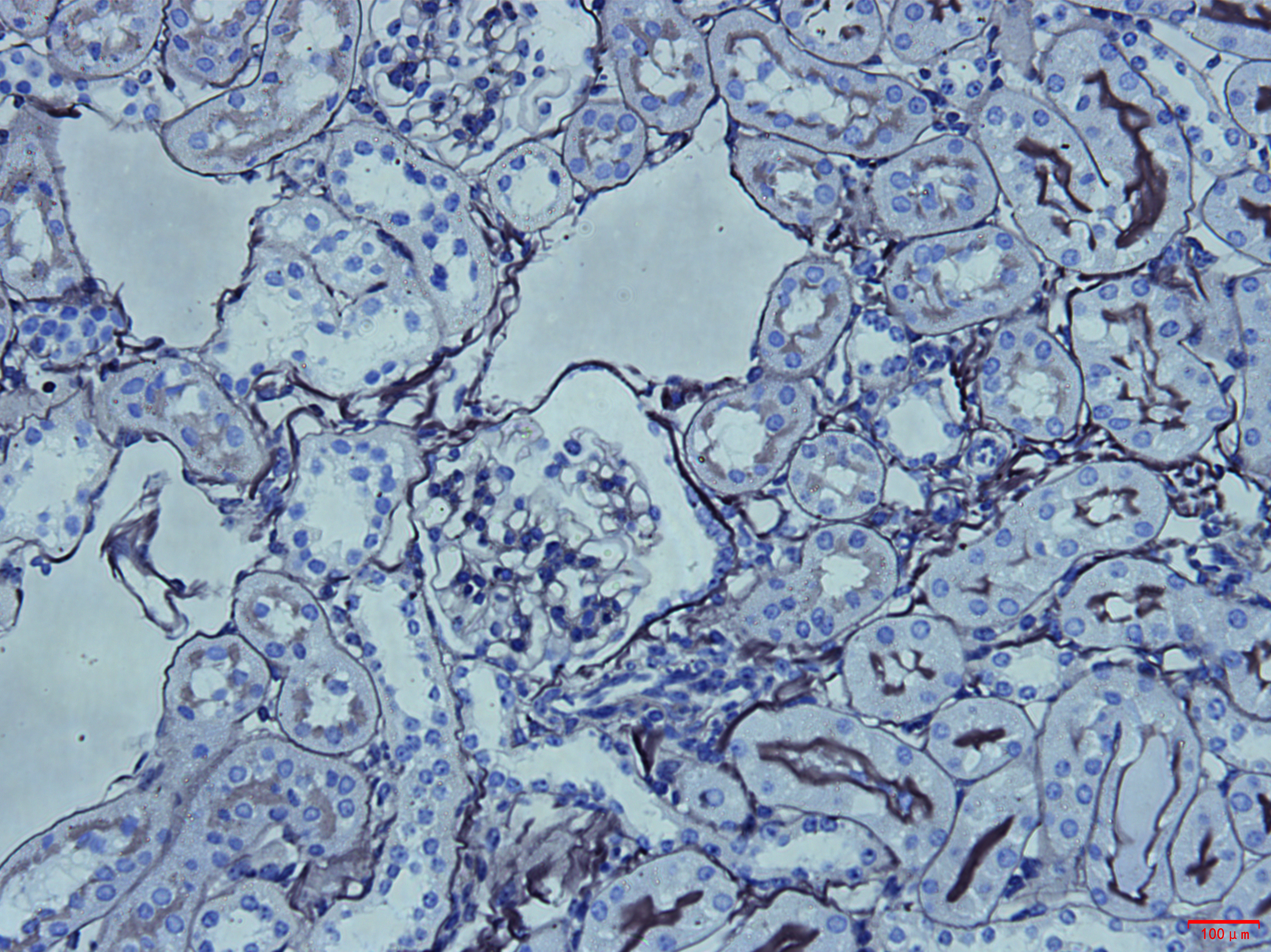

Supplement: S2 File — (ZIP) [file pone.0319605.s002.zip › Fig 2D low.jpg]

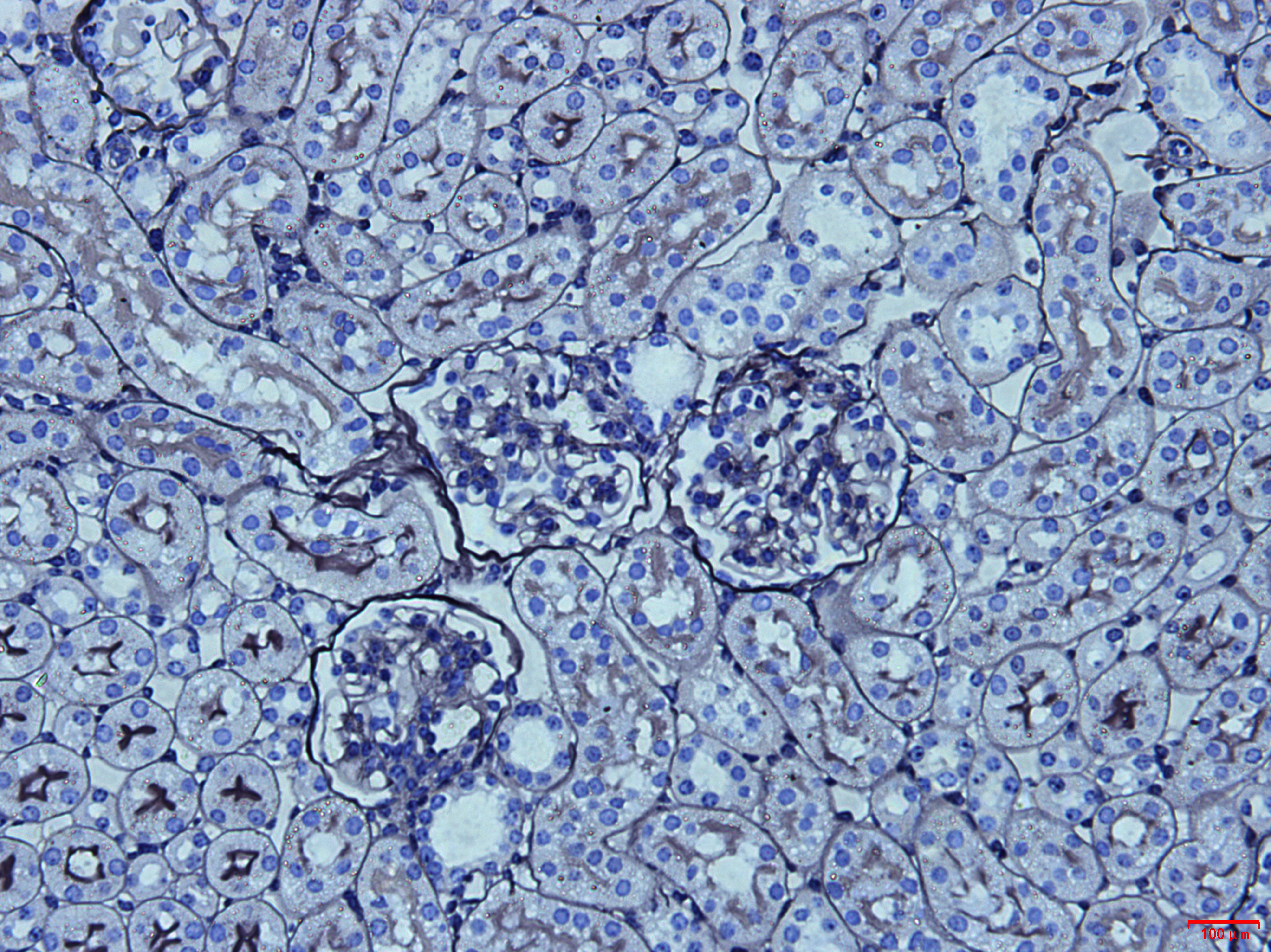

Supplement: S2 File — (ZIP) [file pone.0319605.s002.zip › Fig 2D up.jpg]

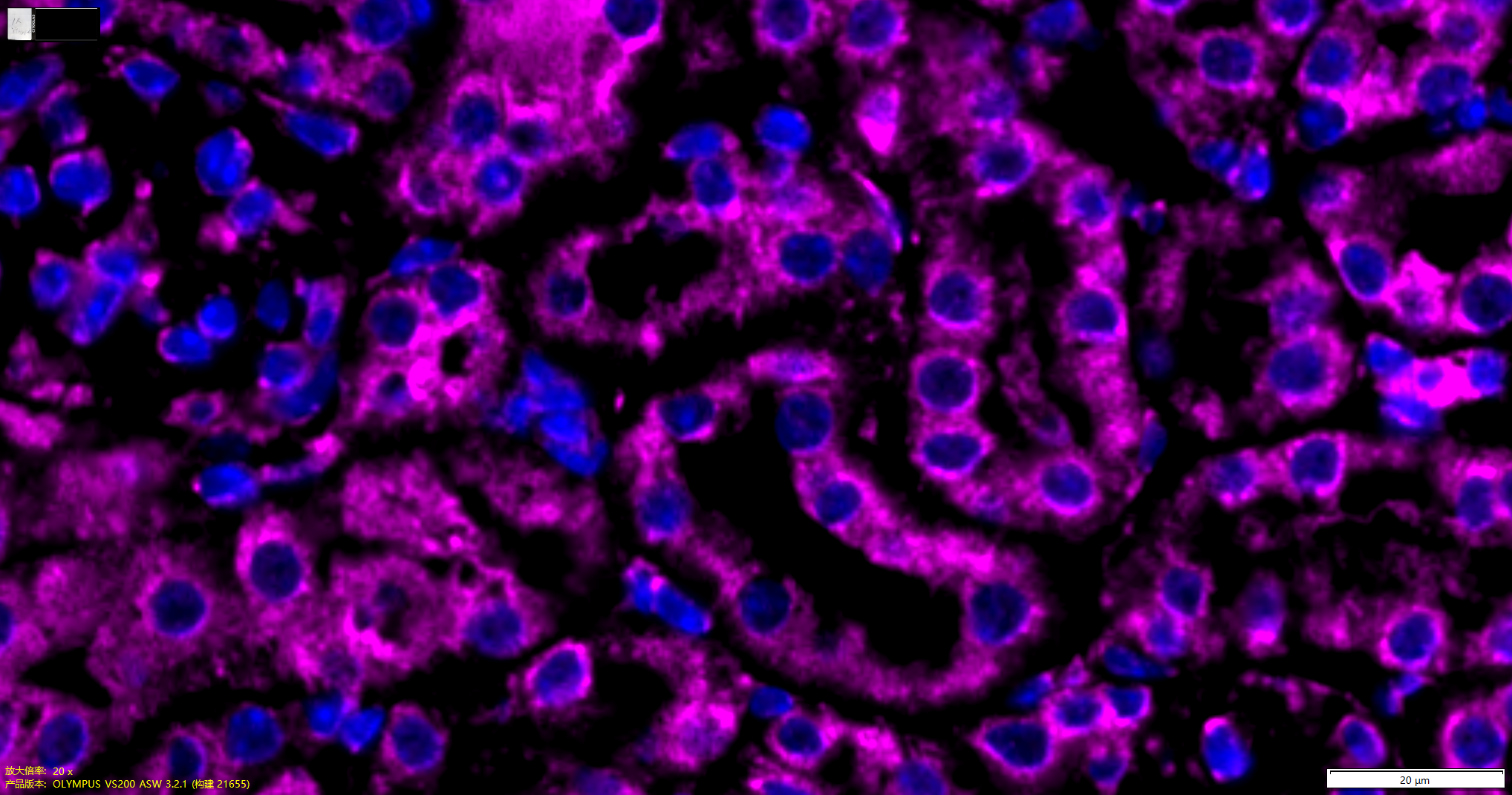

Supplement: S2 File — (ZIP) [file pone.0319605.s002.zip › Fig 2I low merge.tif]

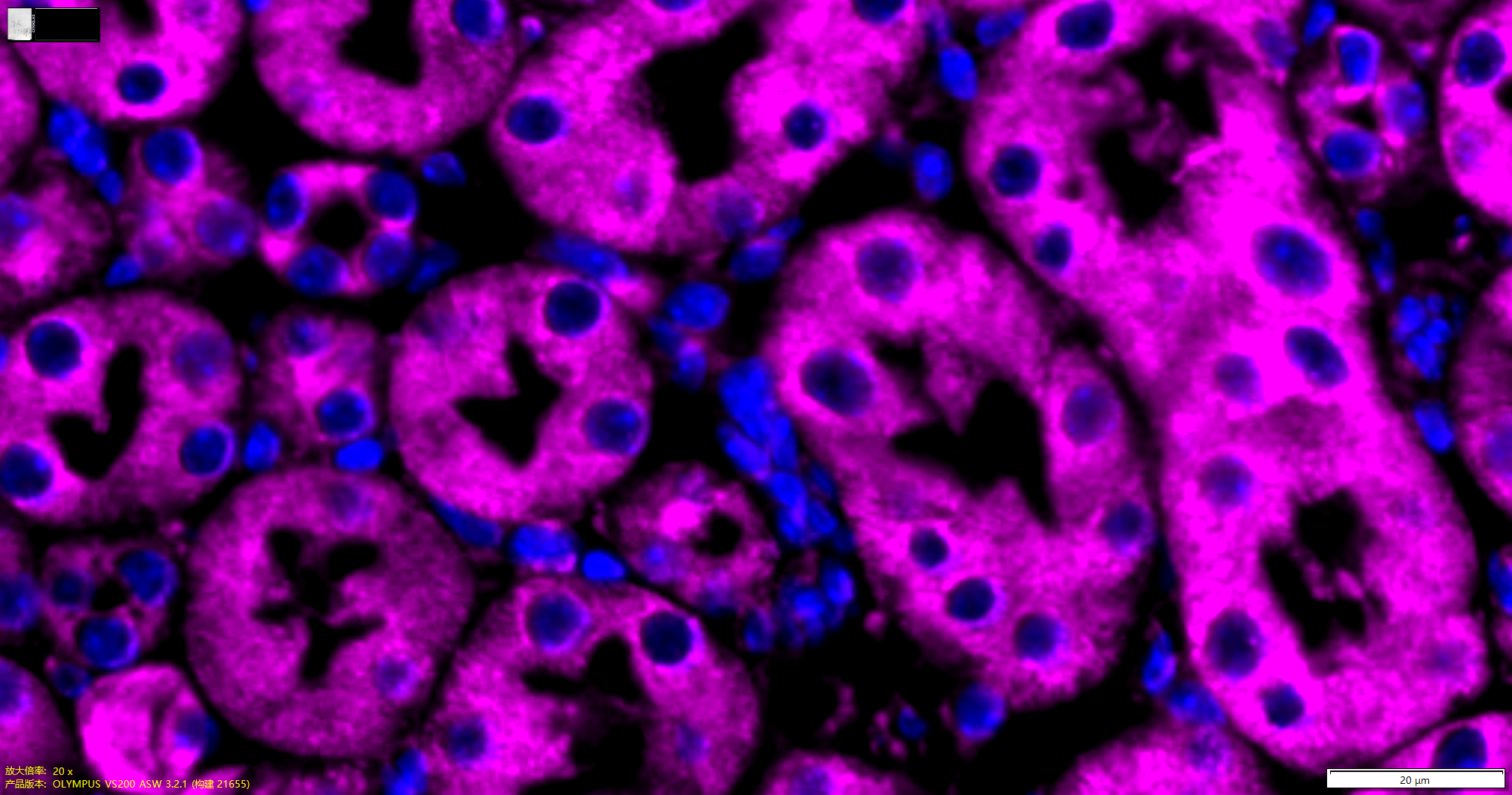

Supplement: S2 File — (ZIP) [file pone.0319605.s002.zip › Fig 2I up merge.tif]

Extended Data Figure 1C original blots

Bcl2 26kDa

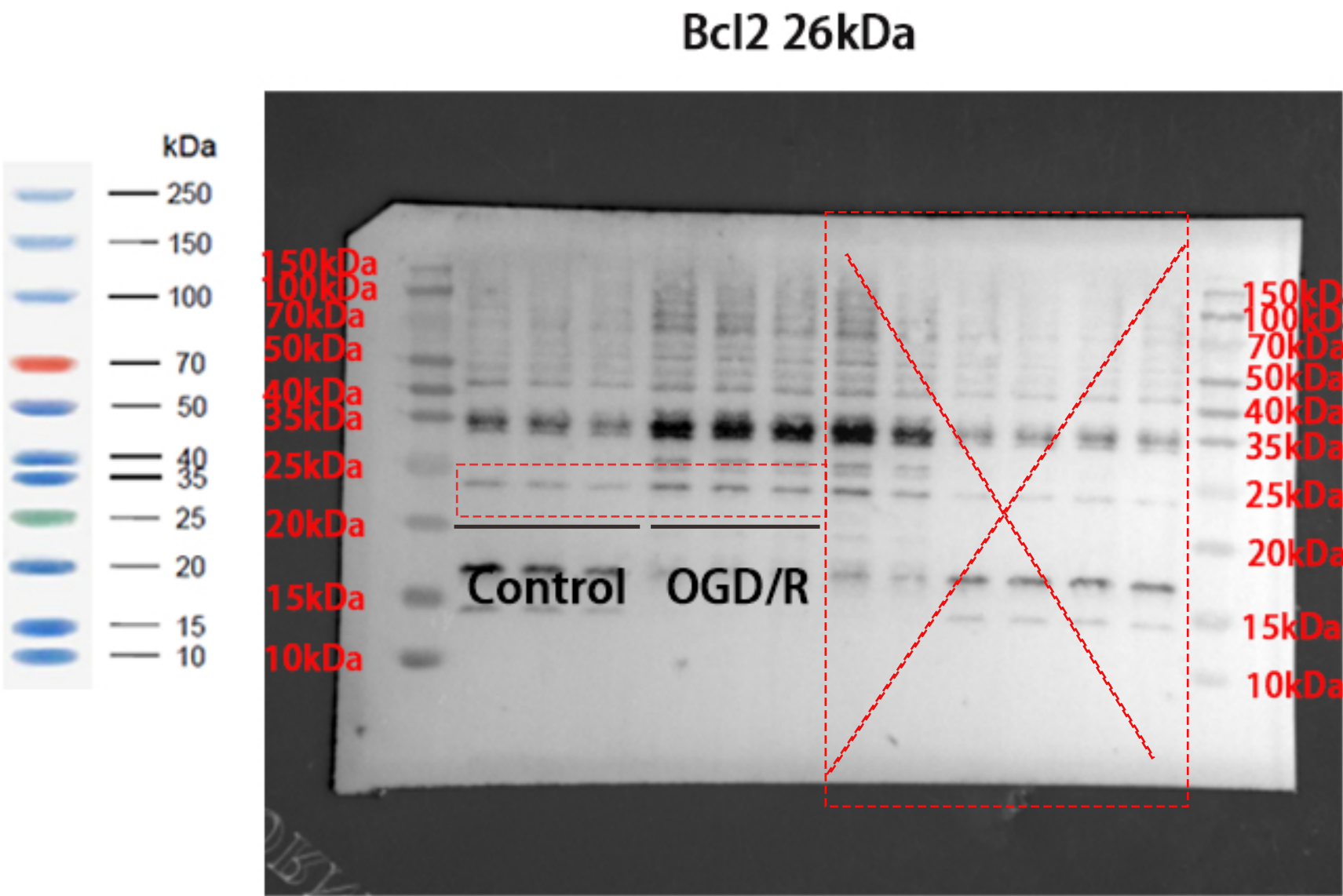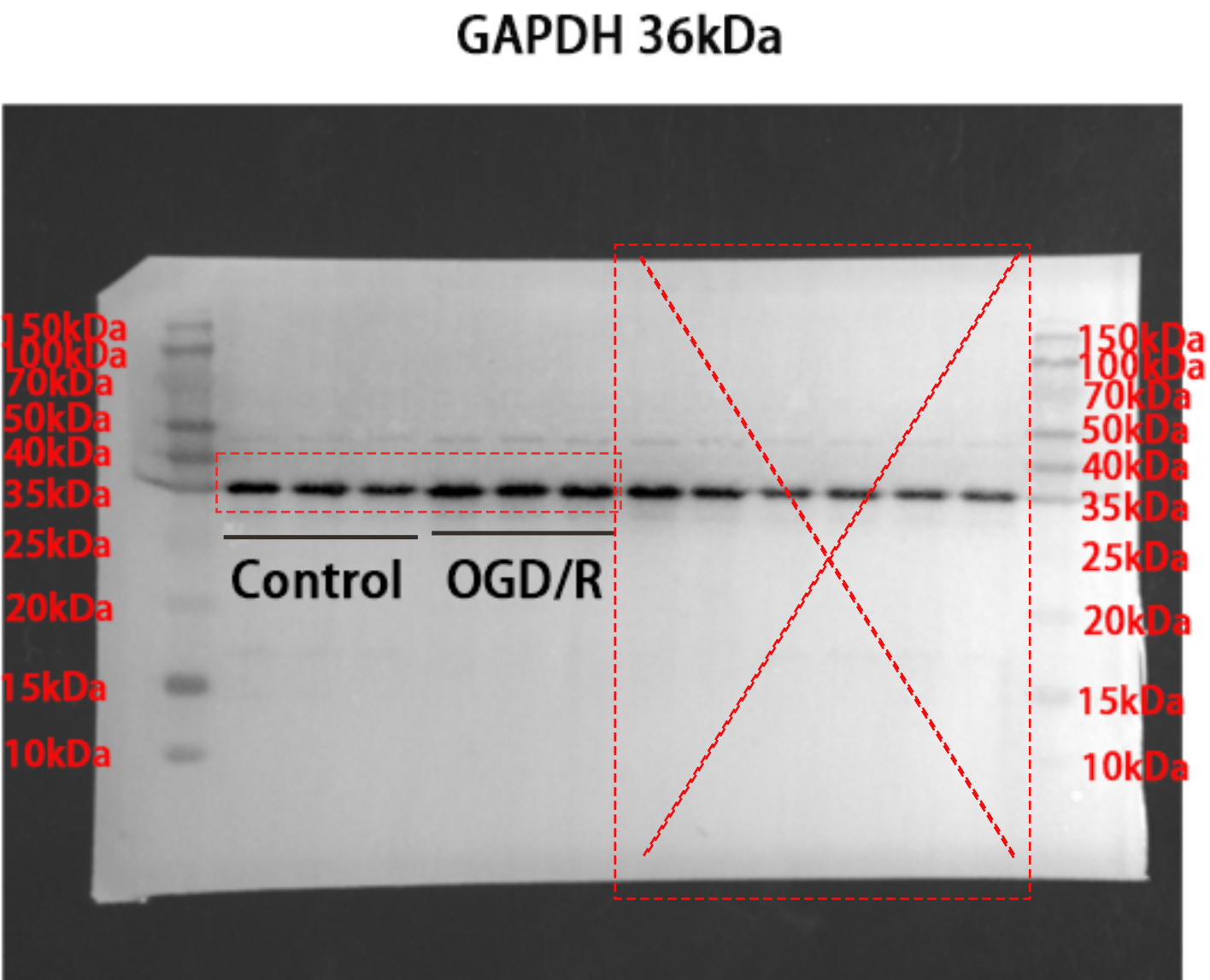

Caspase-3 32kDa

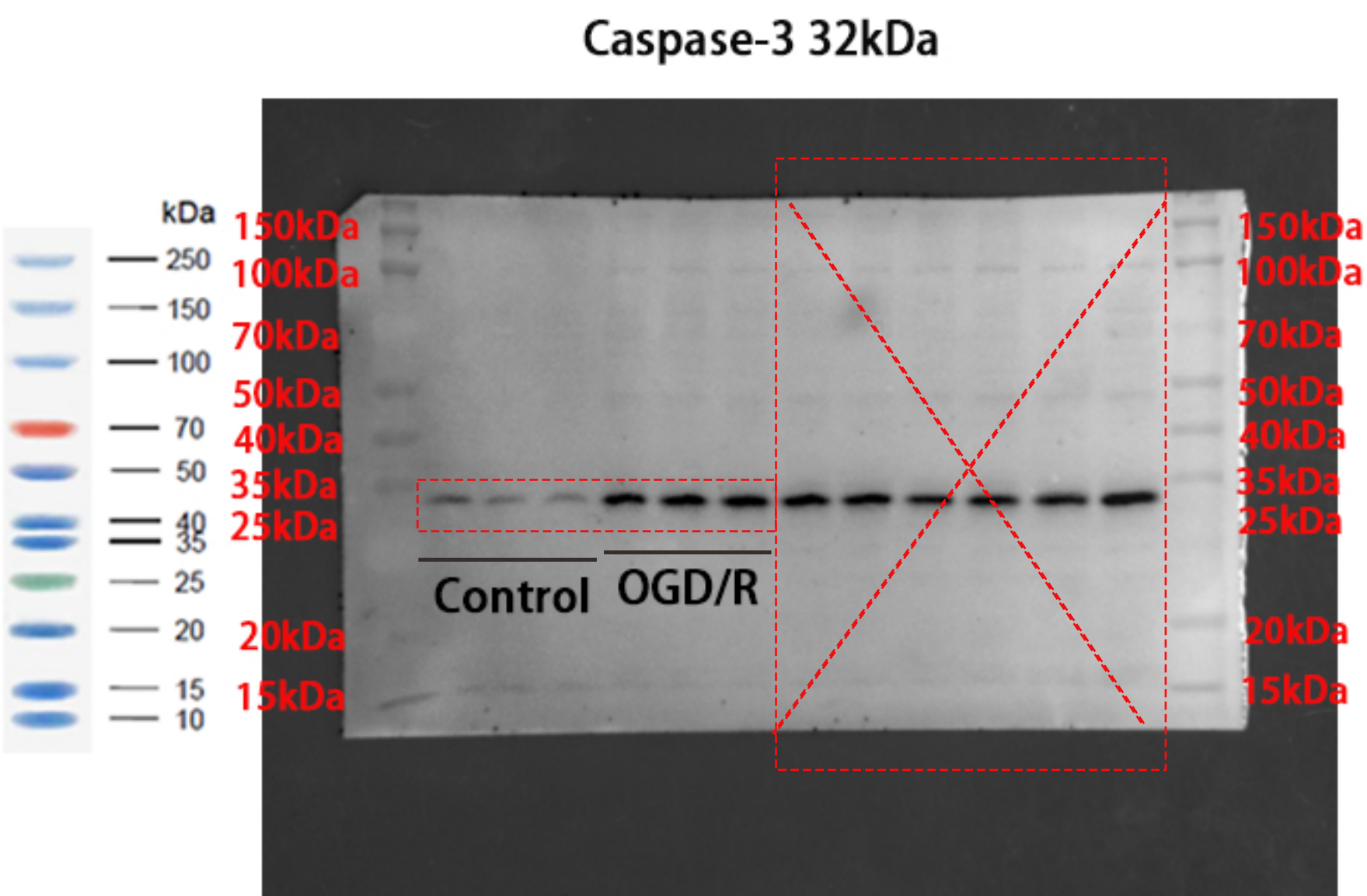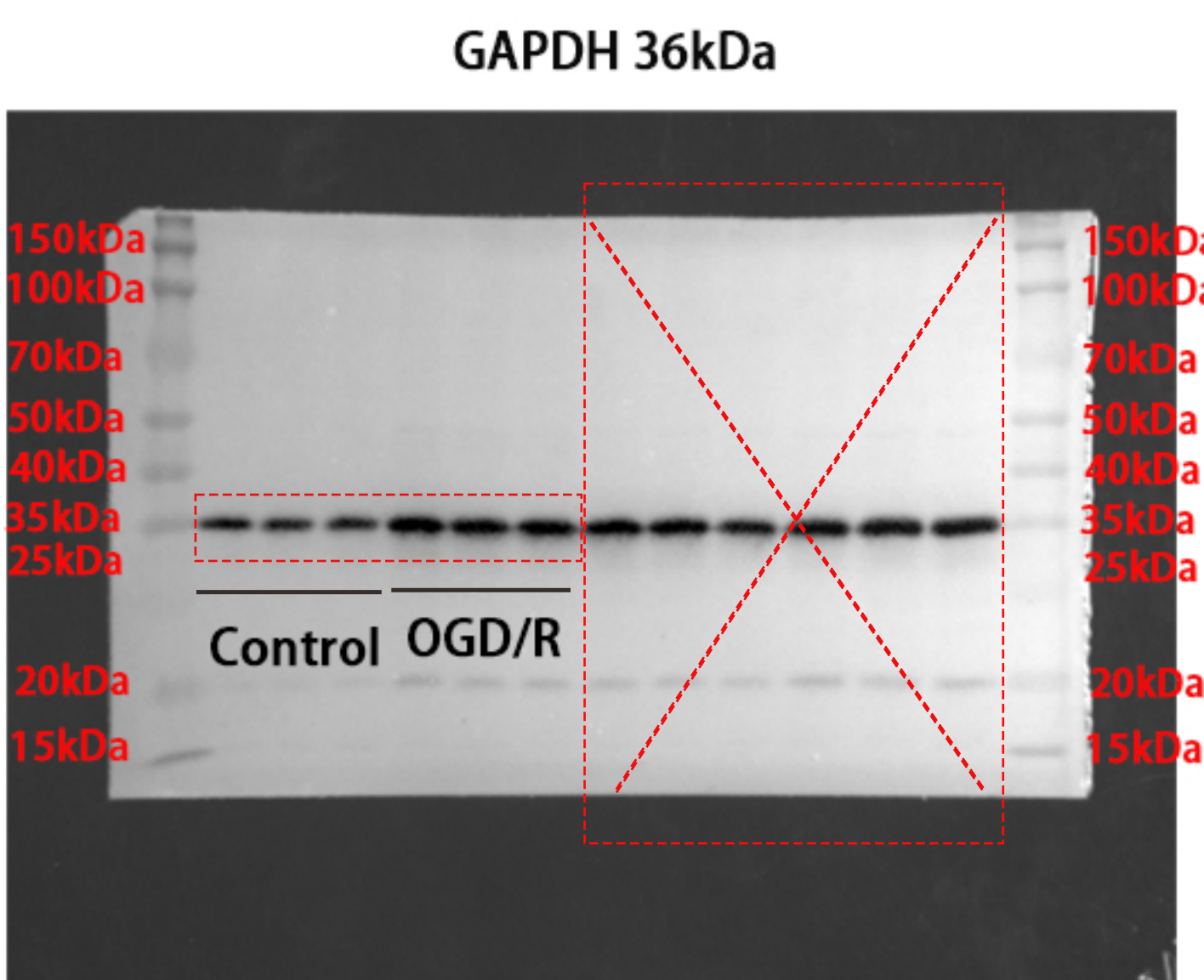

Bax 21kDa

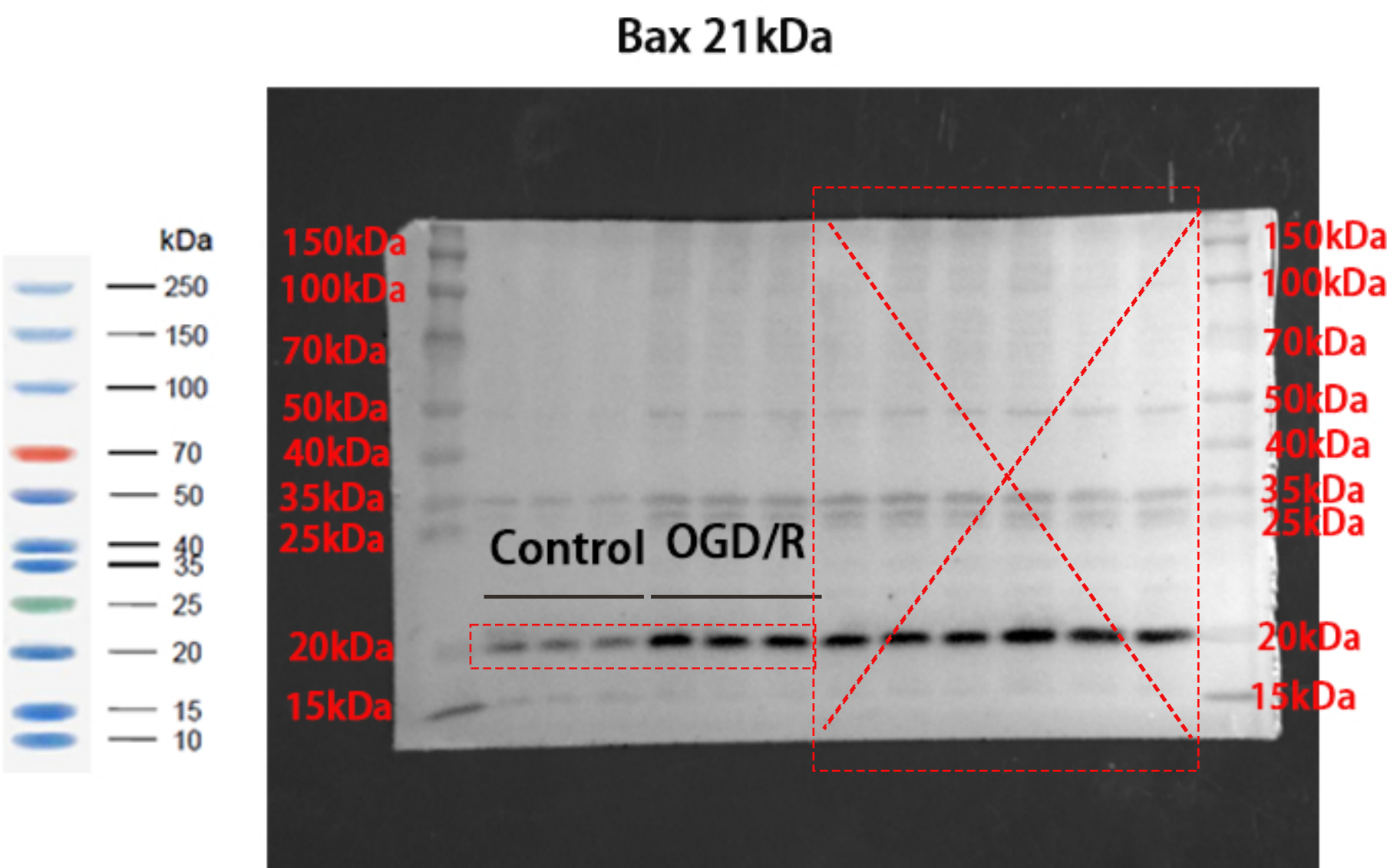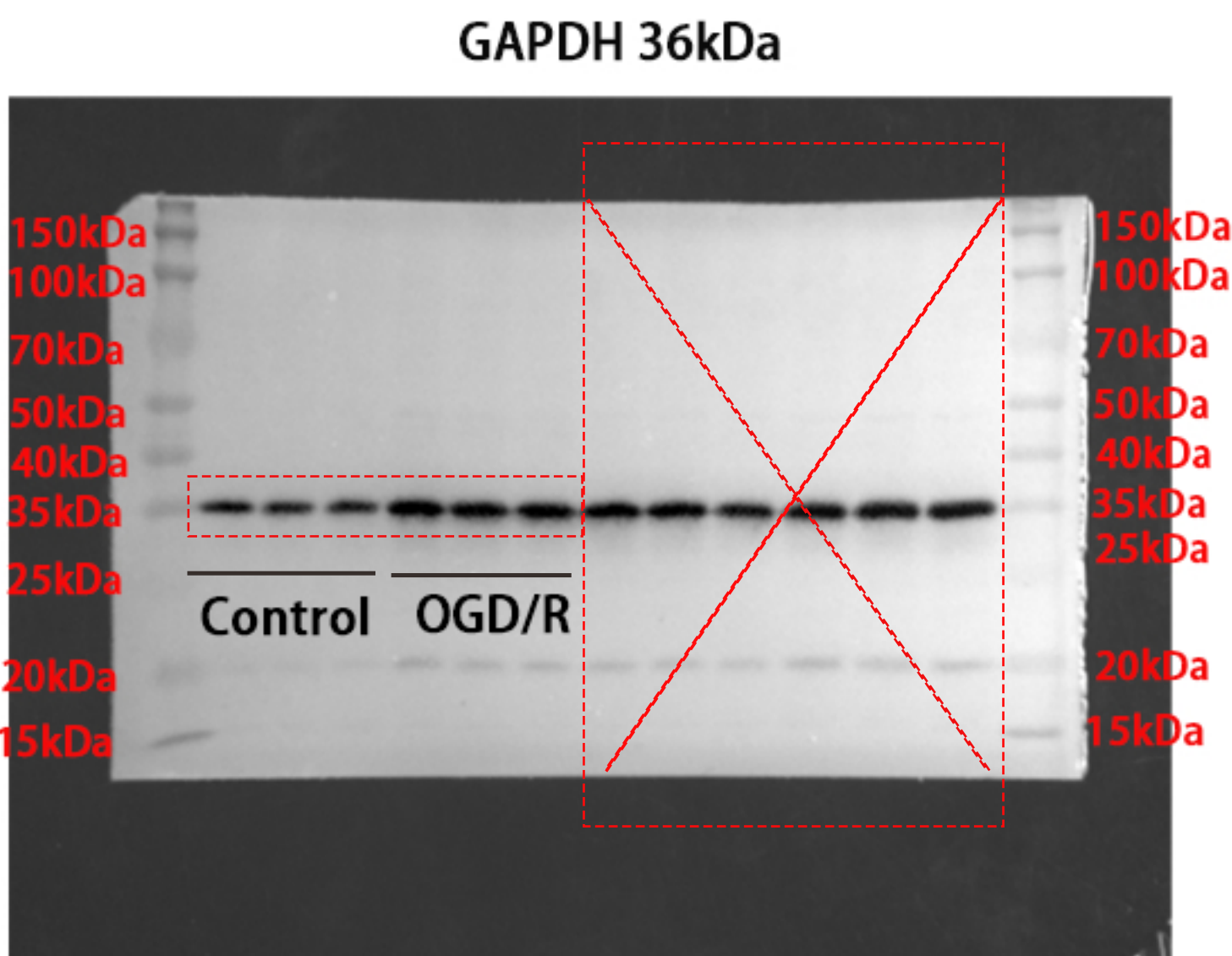

Supplement: S3 File — (ZIP) [file pone.0319605.s003.zip › Fig 1C.pdf]

# Extended Data Figure 1H original blots

YTHDF2 62kDa+ GAPDH 36kDa

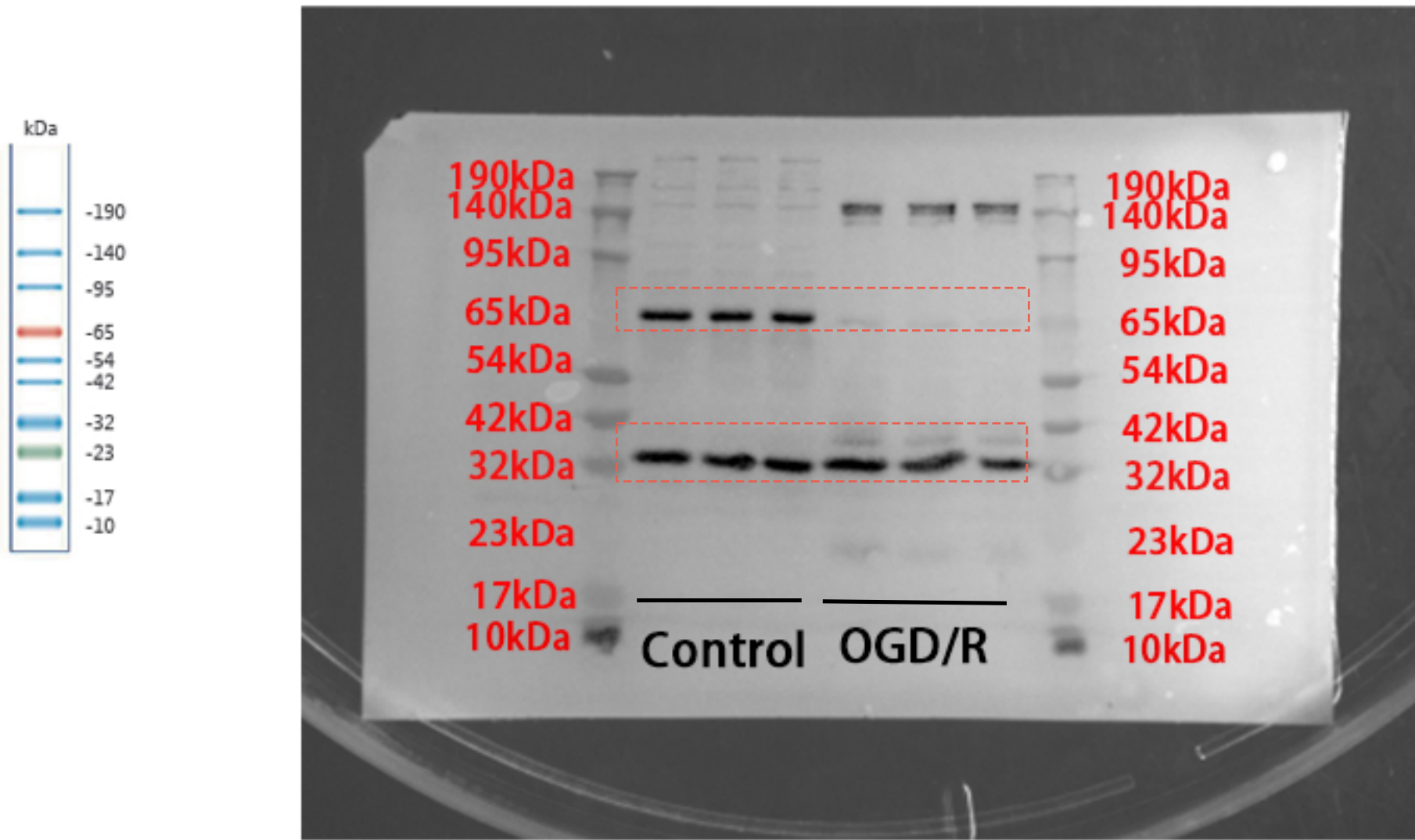

YTHDF2 62kDa

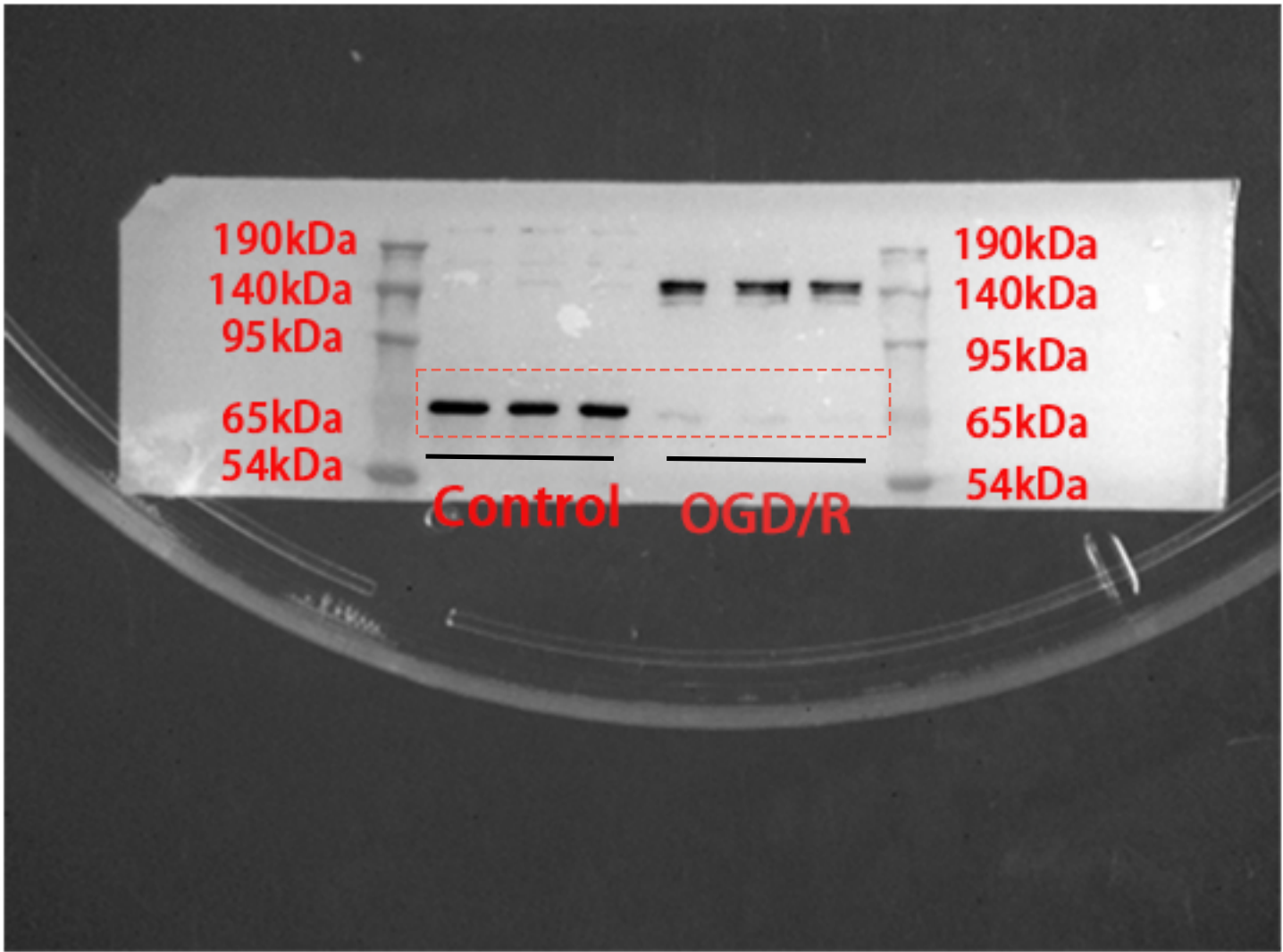

Intact membrane ↑

GAPDH 36kDa

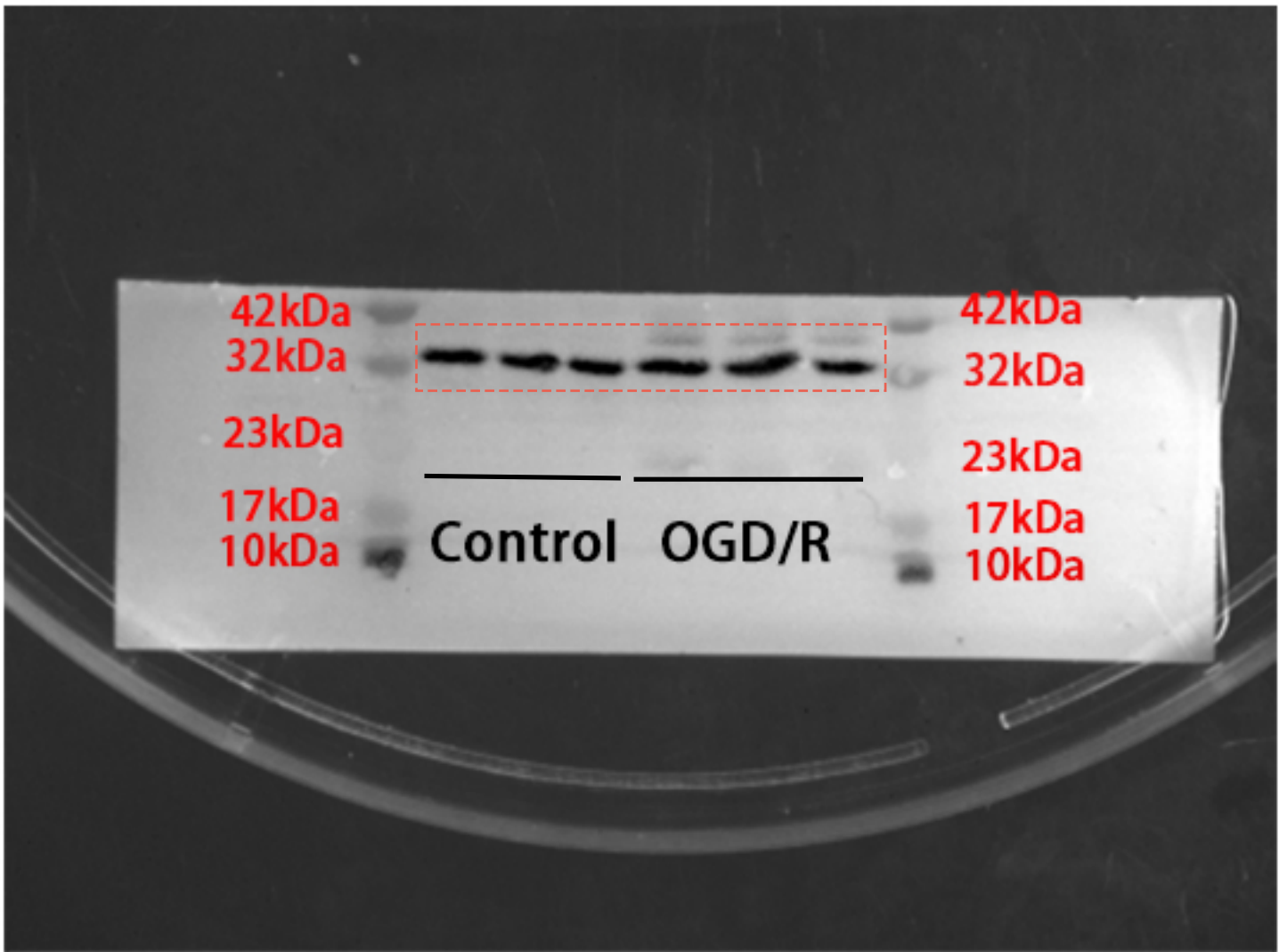

Supplement: S3 File — (ZIP) [file pone.0319605.s003.zip › Fig 1H.pdf]

# Extended Data Figure 2G original blots

YTHDF2 62kDa

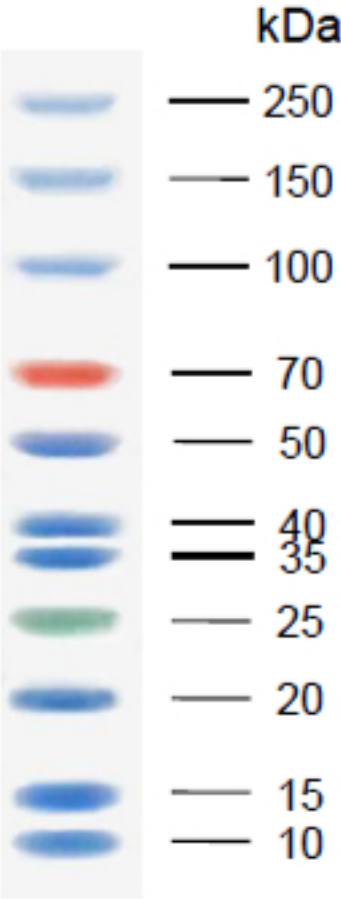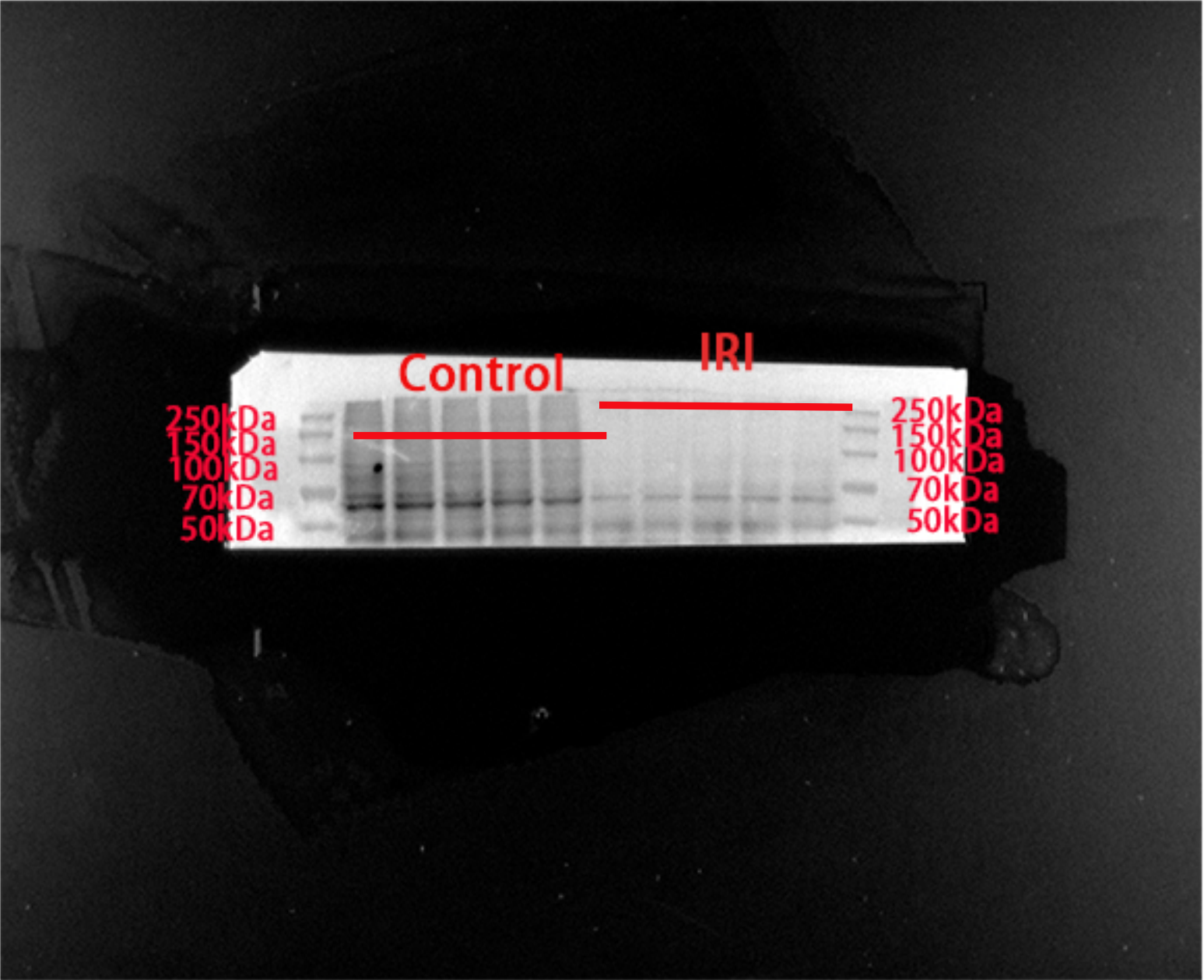

GAPDH 36kDa

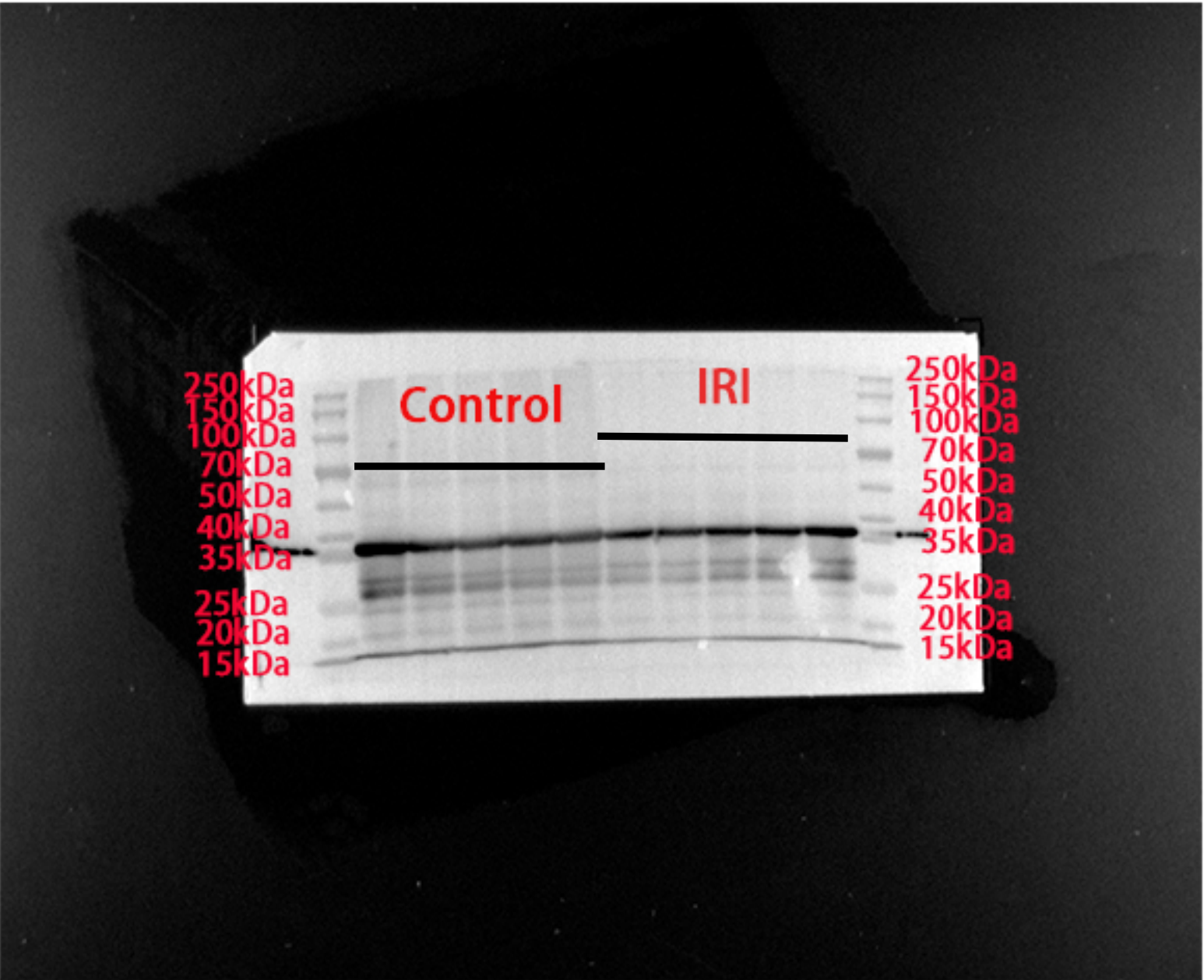

Supplement: S3 File — (ZIP) [file pone.0319605.s003.zip › Fig 2G.pdf]

# Extended Data Figure 3B original blots

YTHDF2 62kDa

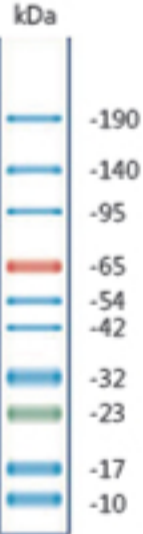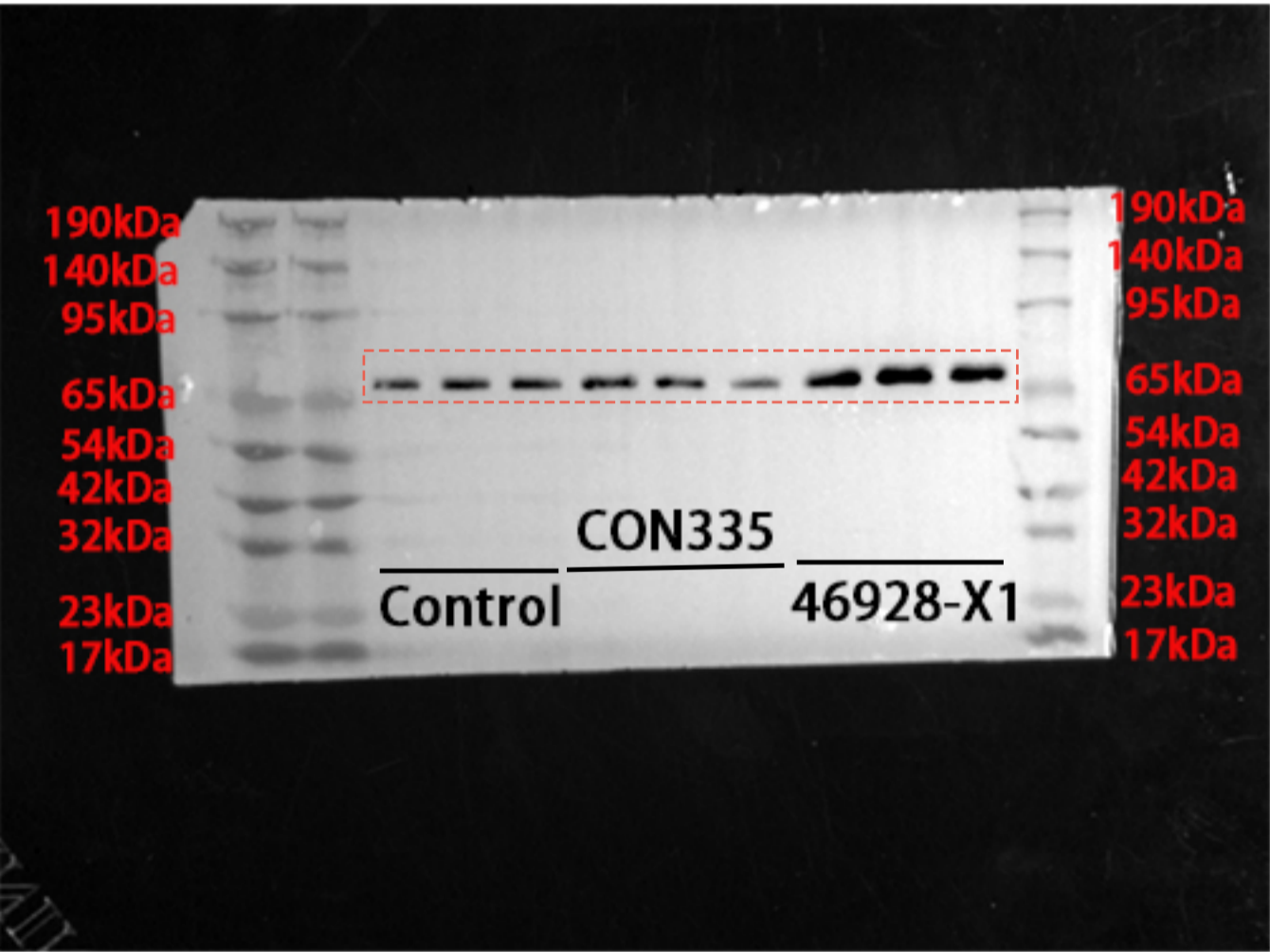

YTHDF2 62kDa+GAPDH 36kDa

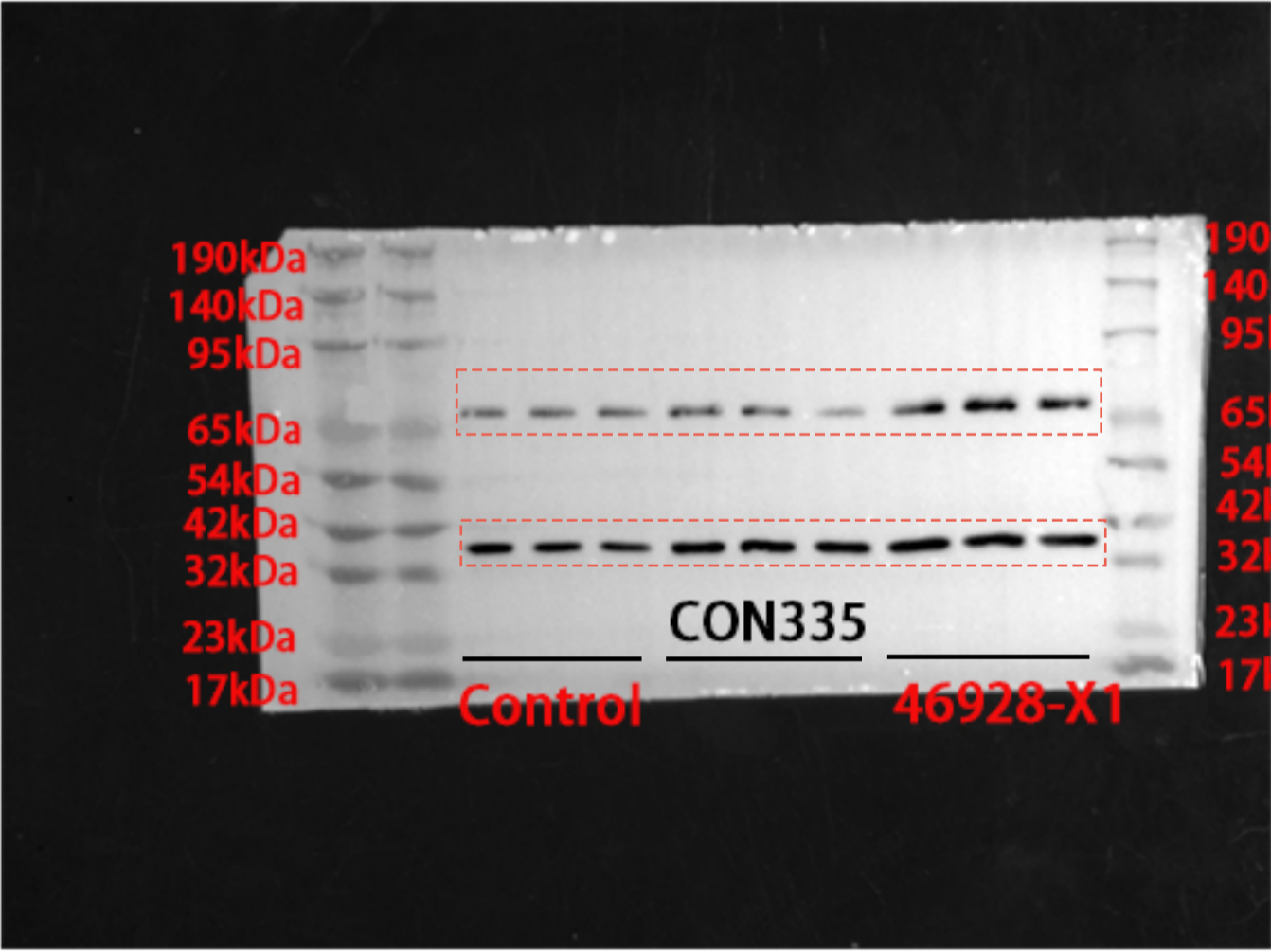

Supplement: S3 File — (ZIP) [file pone.0319605.s003.zip › Fig 3B.pdf]

Extended Data Figure 3F original blots

Bcl2 26kDa

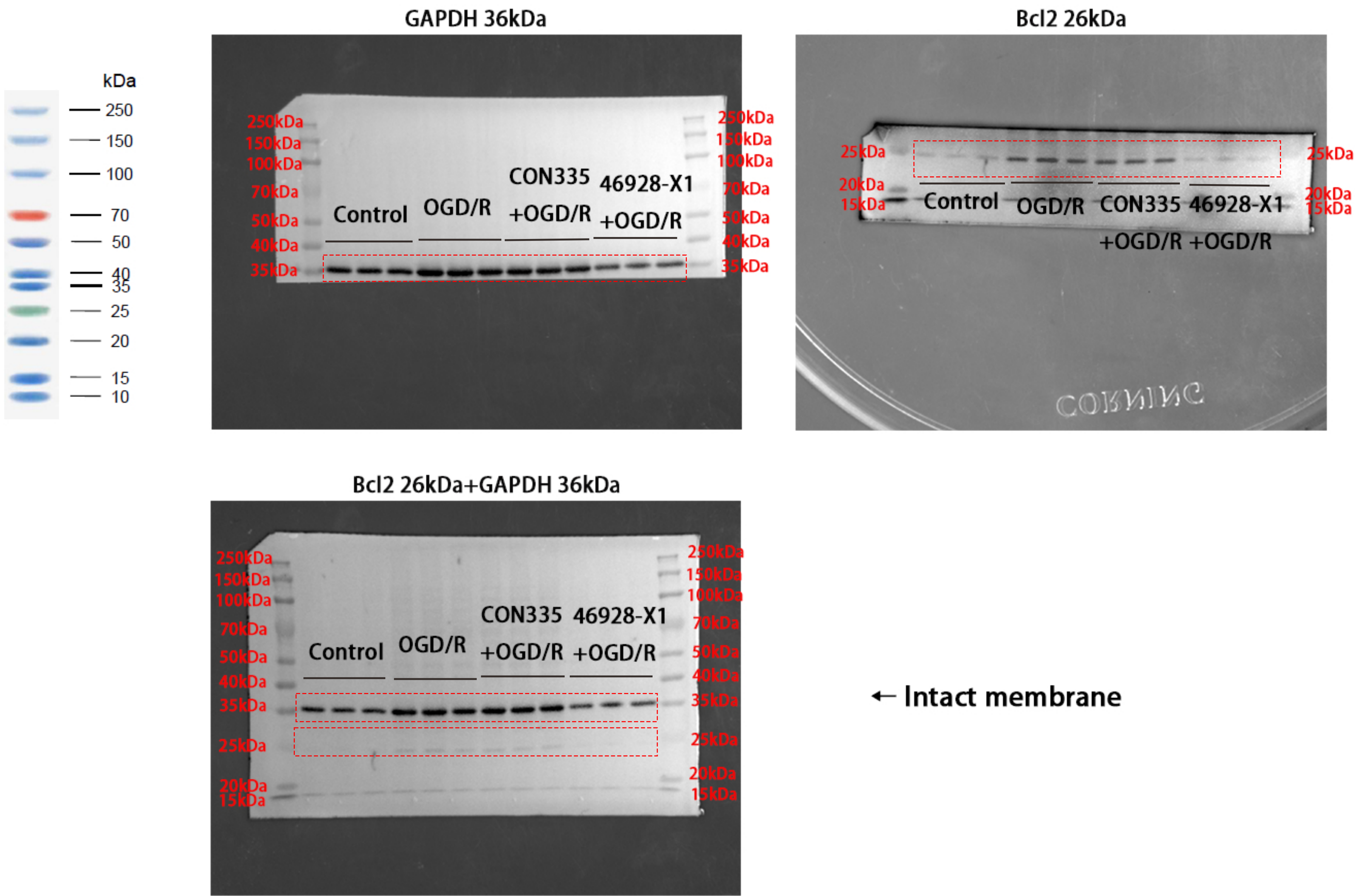

Bax 21kDa

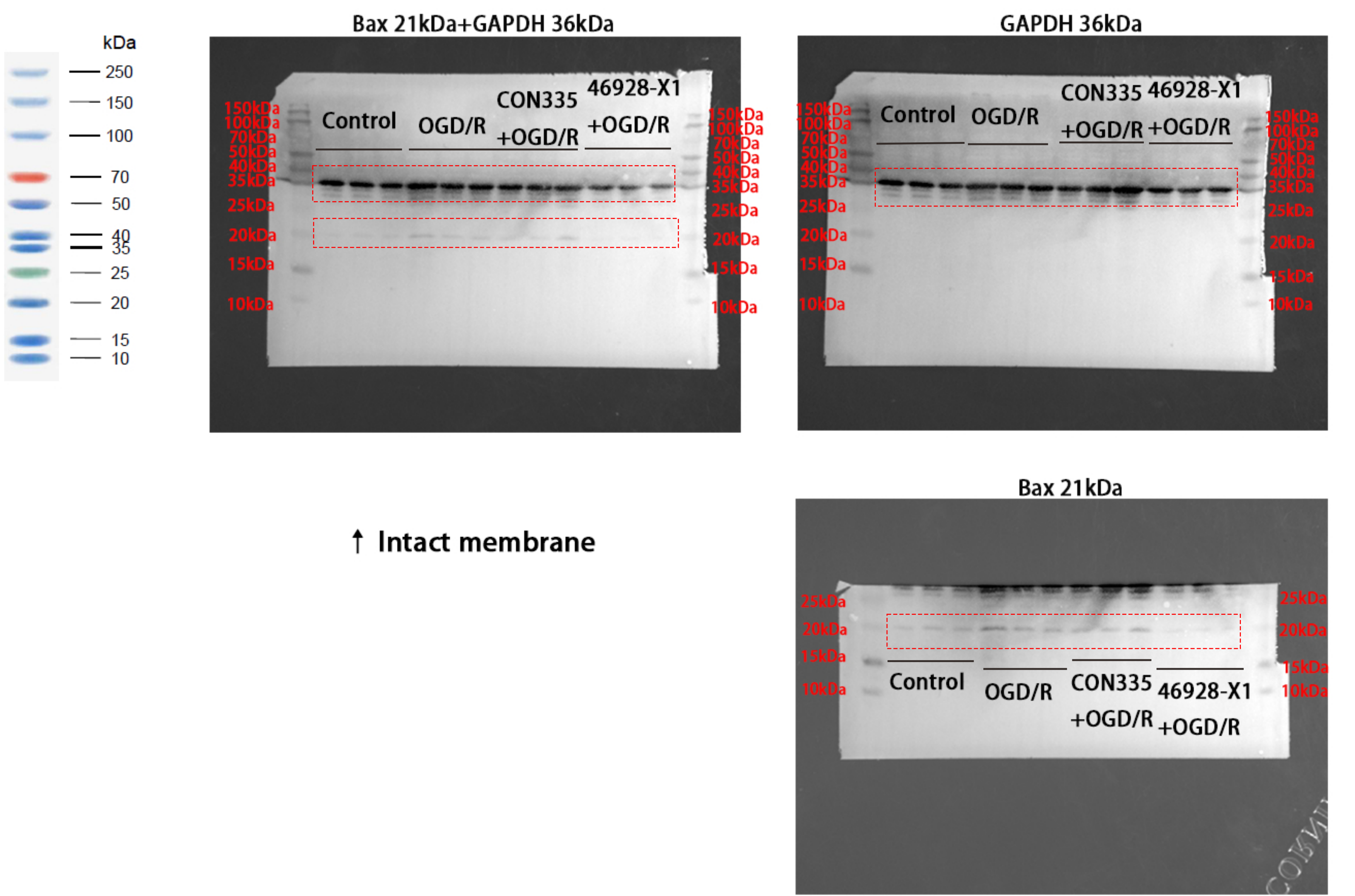

Caspase-3 32kDa

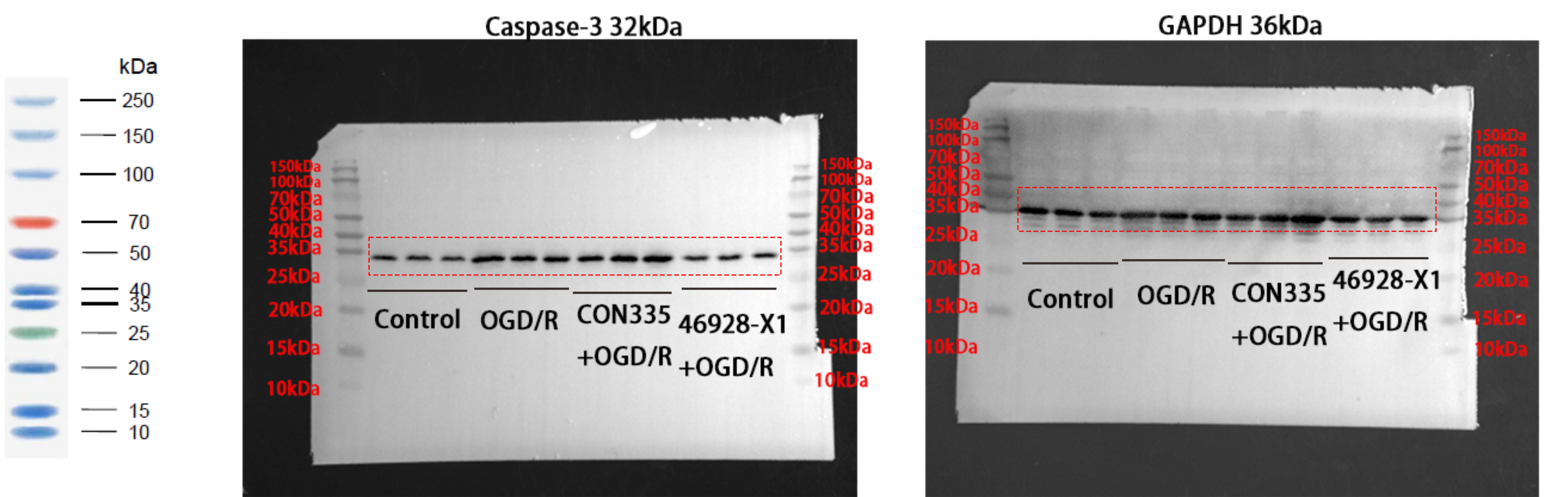

Supplement: S3 File — (ZIP) [file pone.0319605.s003.zip › Fig 3F.pdf]

# Extended Data Figure 4B original blots

YTHDF2 62kDa

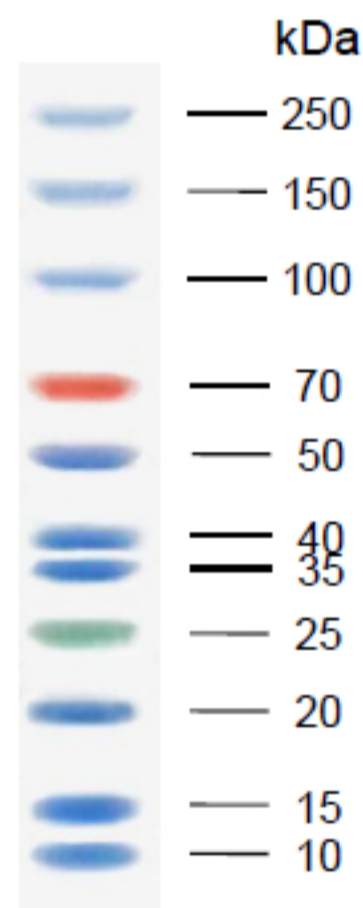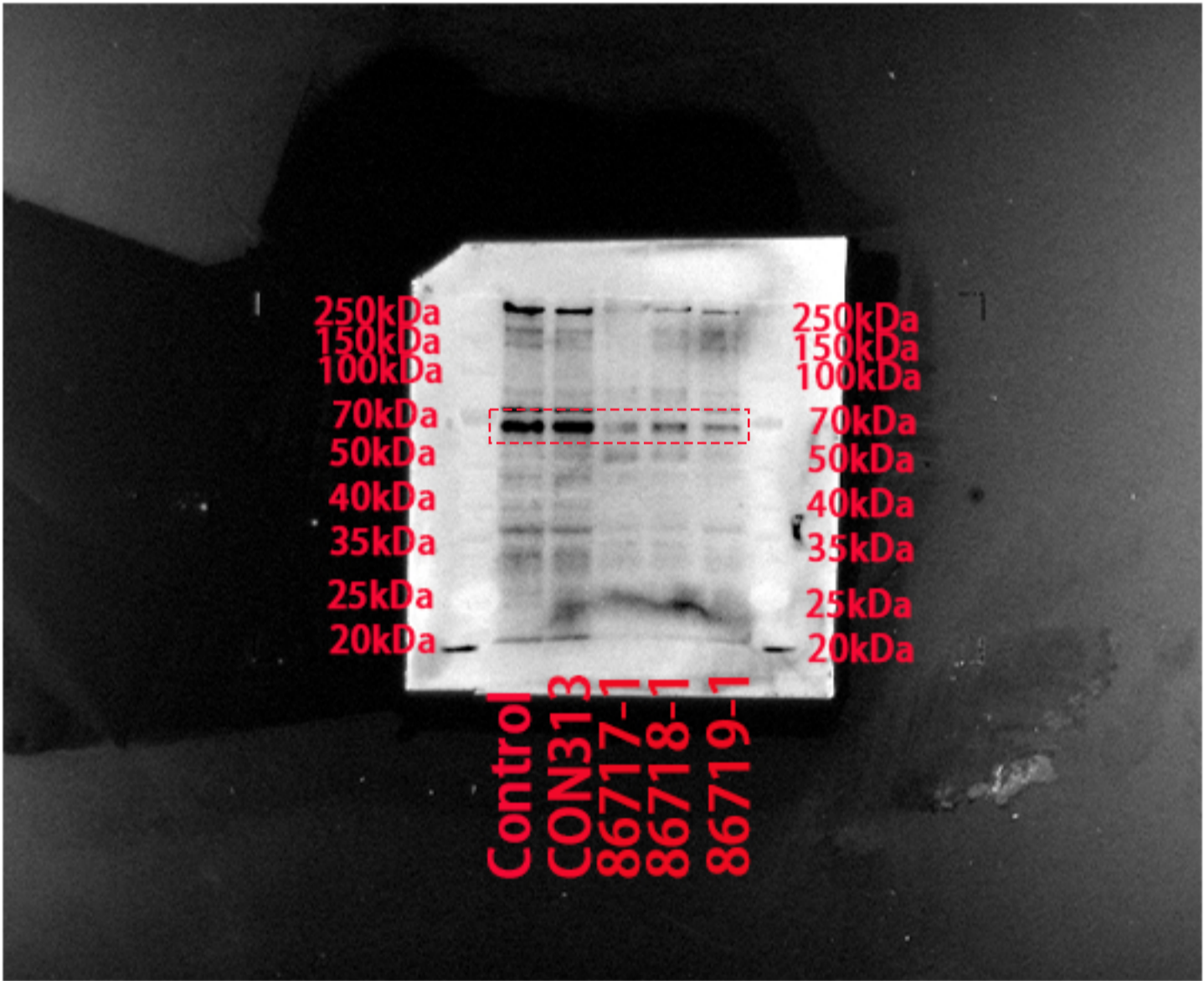

GAPDH 36kDa

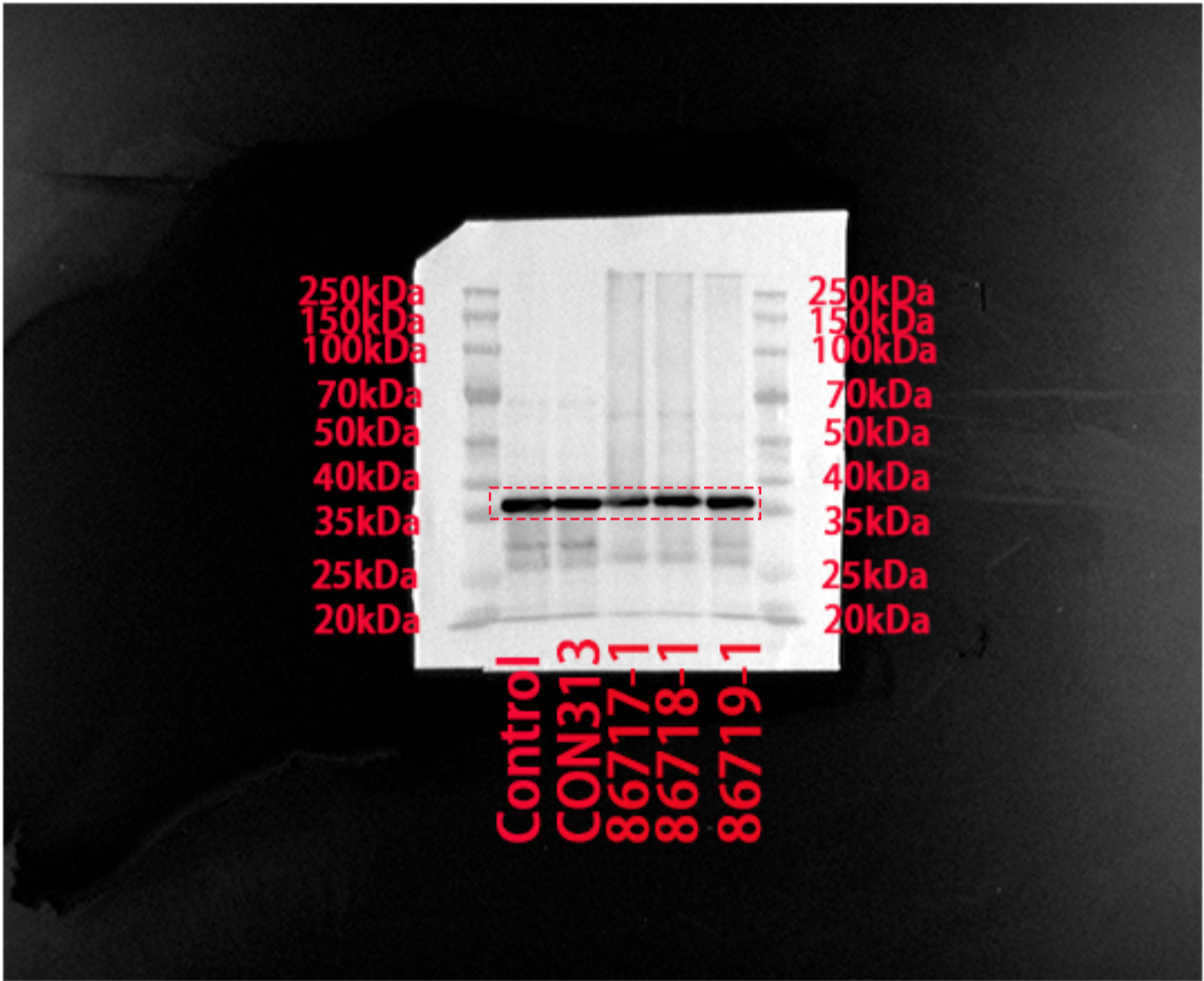

Supplement: S3 File — (ZIP) [file pone.0319605.s003.zip › Fig 4B.pdf]

## Extended Data Figure 5C original blots

YTHDF2 62kDa

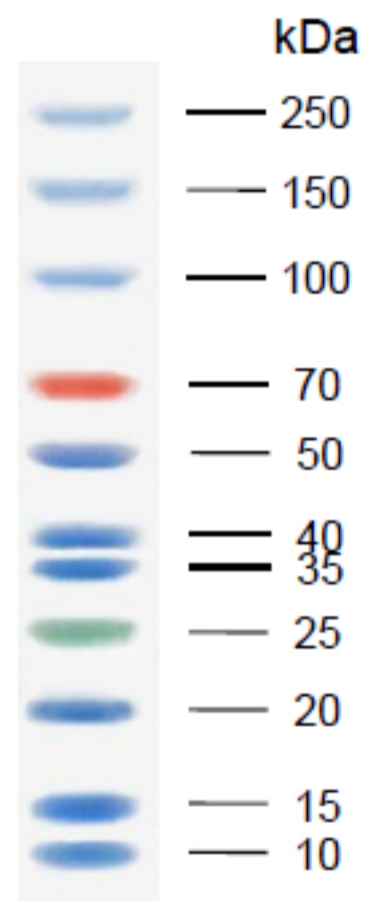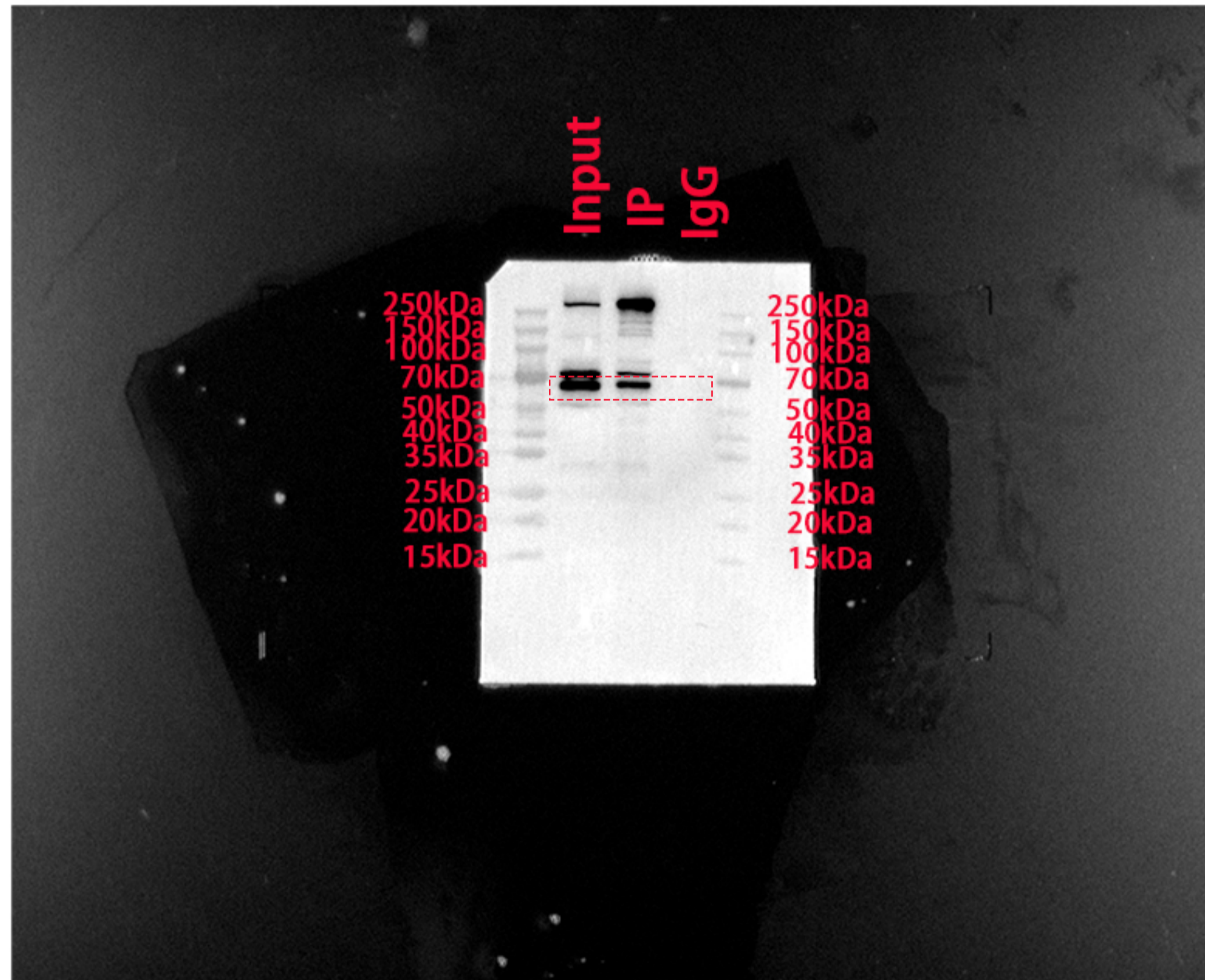

Supplement: S3 File — (ZIP) [file pone.0319605.s003.zip › Fig 5C.pdf]

# Extended Data Figure 5I original blots

## MMP2 72kDa

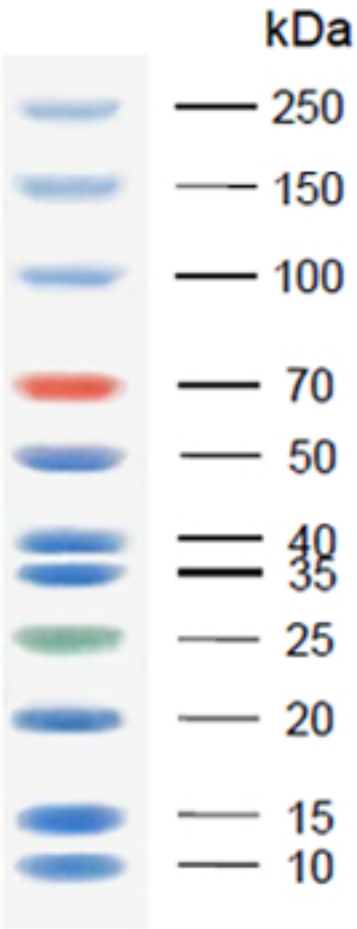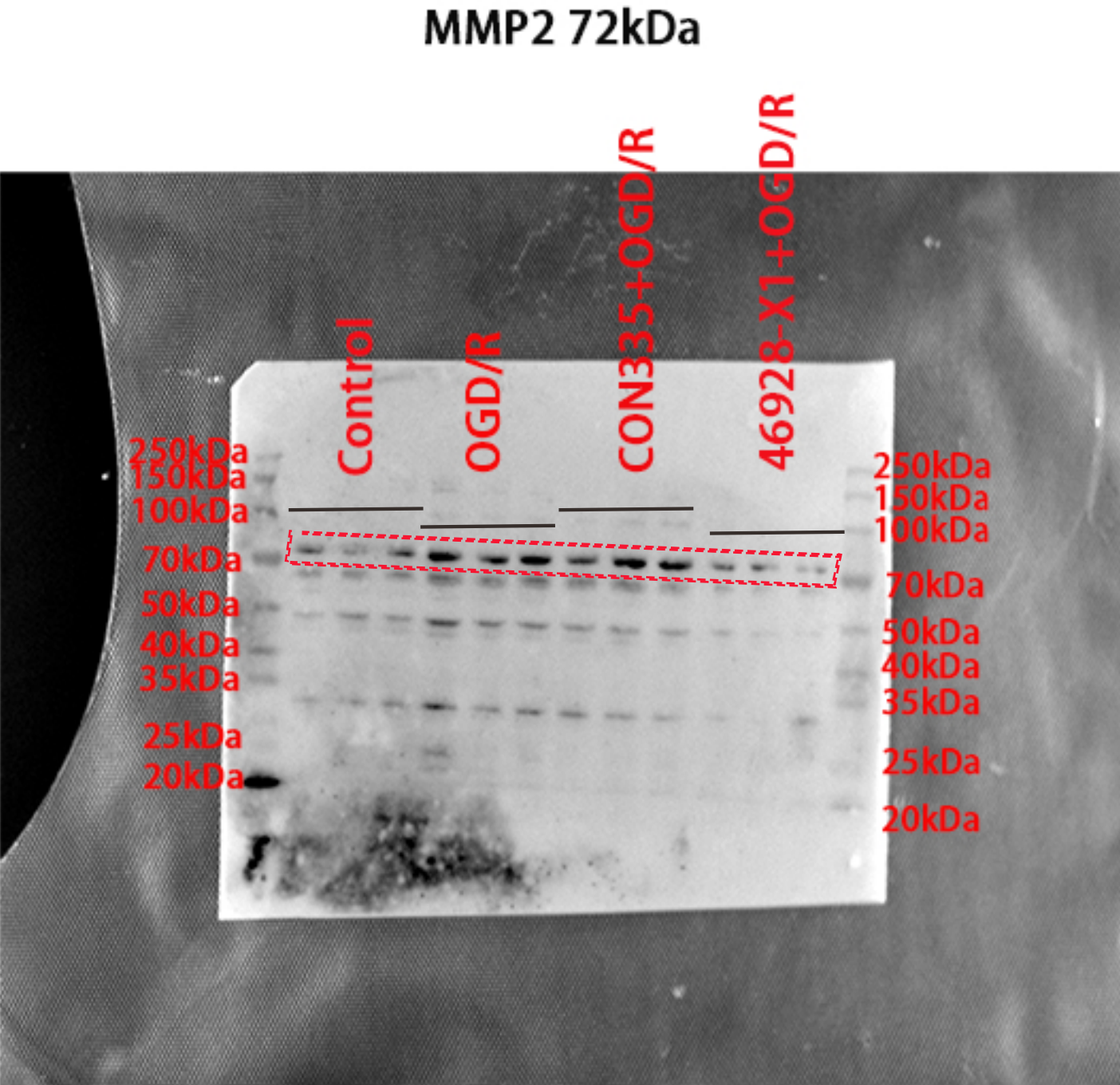

## GAPDH 36kDa

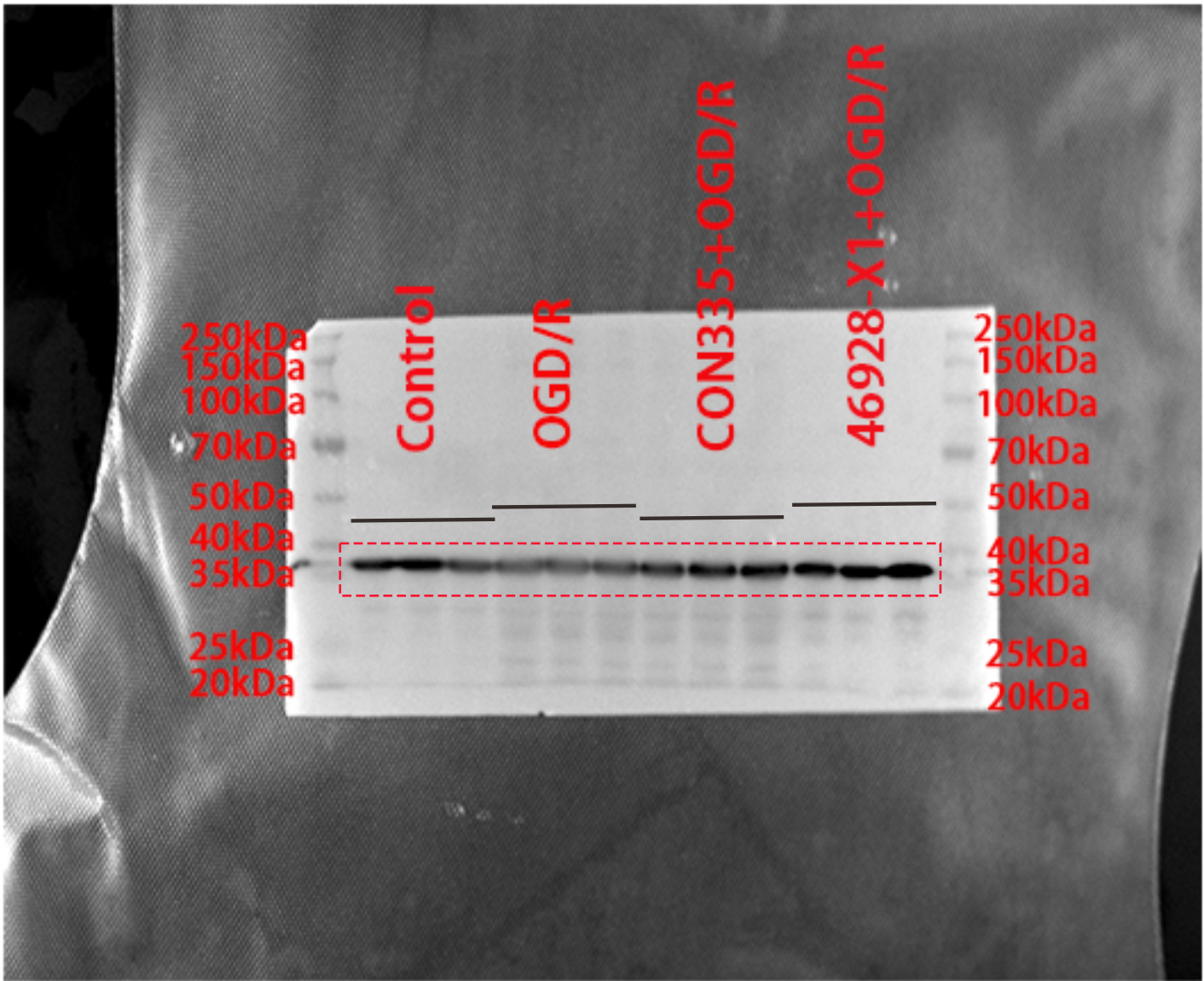

Supplement: S3 File — (ZIP) [file pone.0319605.s003.zip › Fig 5I.pdf]
